# Knowledge, Attitudes, and Practices of Hygiene and the Prevention of Trachoma in the Indigenous Population of the Colombian Amazon Vaupés Department

**DOI:** 10.3390/ijerph20054632

**Published:** 2023-03-06

**Authors:** Julián Trujillo-Trujillo, Mónica Meza-Cárdenas, Sol Beatriz Sánchez, Sara Milena Zamora, Alexandra Porras, Clara Beatriz López de Mesa, Luz Mery Bernal Parra, María Consuelo Bernal Lizarazú, Hollman Miller, Juan Carlos Silva

**Affiliations:** 1 Ministry of Health and Social Protection, Bogotá 110311, Colombia; 2 Escuela de Ciencias de la Salud—ECISA, Universidad Nacional Abierta y a Distancia, UNAD, Bogotá 111511, Colombia; 3 Grupo de Medicina Comunitaria y Salud Colectiva, Maestría en Epidemiología, Facultad de Medicina, Universidad El Bosque, Bogotá 110111, Colombia; 4 Escuela Superior de Oftalmología, Instituto Barraquer de América, Bogotá 110321, Colombia; 5 Department of Vaupés, Secretariate of Health, Mitú 970001, Colombia; 6 Independent Researcher, Bogotá 110111, Colombia

**Keywords:** SAFE strategy, indigenous population, face washing

## Abstract

The Colombian program to end trachoma implements the component F of the SAFE strategy in the Vaupés department of the Amazon rainforest. Cultural, linguistic, and geographical barriers and the coexistence of an ancestral medical system demand the technical and sociocultural adaptation of this component. A cross-sectional survey combined with focus-group discussions to understand the knowledge, attitudes, and practices of the indigenous population related to trachoma was conducted in 2015. Of the 357 heads of households that participated, 45.1% associated trachoma with a lack of hygiene, and 94.7% associated the concept of hygiene with taking one or more body baths per day, using commercial or handcrafted soap. In total, 93% reported cleaning their children’s faces and eyes more often when they have conjunctivitis, but 66.1% also did this with clothes or towels in use, and 52.7% of people shared towels; in total, 32.8% indicated that they would use ancestral medicine to prevent and treat trachoma. The SAFE strategy in Vaupés requires an intercultural approach to facilitate stakeholder support and participation to promote general and facial hygiene, washing clothes with soap, and not sharing towels and clothes to dry and clean children’s faces for effective and sustainable elimination of trachoma as a public health problem. This qualitative assessment facilitated an intercultural approach locally and in other Amazonian locations.

## 1. Introduction

Trachoma is an infectious disease and is the most important infectious cause of blindness. According to the WHO, it is a public health problem in 44 countries, with 136 million people living in trachoma-endemic areas and approximately 1.9 million having developed blindness or visual impairment as a result [1]. Clinically, it is chronic and recurrent keratoconjunctivitis caused by the serotypes A, B, Ba, and C of *Chlamydia trachomatis* that affect people living in areas with limited access to safe drinking water, basic sanitation, and conditions of poverty; it is endemic in many regions of the world, including some Latin American countries [2]. It is preventable and possible to eliminate as a public health problem through the application of the SAFE strategy recommended by the World Health Organization (WHO), in which “S” refers to surgery for trachomatous trichiasis, “A” refers to mass administration of azithromycin, “F” refers to facial cleanliness to reduce bacterial transmission, and “E” refers to environmental improvement, particularly improved access to water and sanitation [3]. Hygiene improvements led to the elimination of trachoma in European countries before the development of the current antibiotics [4].

Vaupés is one of the Amazonian departments with endemic trachoma, located in the Amazonian region on the border with Brazil; it was the first to report this disease in Colombia [5].

The prevalence of trachomatous inflammation—follicular (TF) in Vaupés was 23% (95% CI, 21.9–24.3) in children aged one to nine years, and the frequency of children with secretions in the facial area was 53.6%, measured in 2012 and 2013 [6]. A first impact survey in the eastern district of Vaupés, not yet published, showed a TF of 11%, which exceeded the threshold of 5% defined by the WHO to categorize it as a public health problem [7]. Vaupés has 43,665 inhabitants, one of the lowest population densities in Colombia, with less than one person per square kilometer; the majority are concentrated in Mitú, its capital, with the rest of the population settled in 220 rural communities. The department has a public hospital in Mitú, two health centers (with a doctor, a nurse, and a nursing assistant) in the municipalities of Carurú and Taraira, and 17 health posts in the rural communities, in which indigenous health assistants perform some basic care, serving one or two surrounding communities [8,9].

The rural communities in which the population eligible for the implementation of the SAFE strategy lives in Vaupés are accessible from Mitú mainly by air or several days travel by river; numerous waterfalls and drops in the water flow of the rivers at certain times of the year impede the navigability, leading to geographic isolation of the indigenous people. This situation contributes to preserving their ancestral traditions; however, there is limited access to health services, a poor health infrastructure, and limited availability of health workers, and the health services have high costs for the government.

Vaupés has cultural and linguistic diversity, with 41 tribes speaking more than 30 languages, who use ancestral health medical systems led by traditional healers to prescribe diets, plants, and magical religious resources to restore health and prevent disease. The *Payé* or shamans are the main spiritual leaders, with spiritual powers to guide people, harmonize energies, and seek a balance between humans and nature; the *Cumú* (herbal doctor) and the elders also advise on the prevention and treatment of different diseases [10]. Despite their own ancestral medical system, the Amazonian indigenous people and the traditional healers recognize and accept allopathic medical systems and sometimes combine both, which is the result of a long and permanent intercultural dialog promoted by national and local governments and health authorities [11].

The health system has financed the development of surgeries for trachomatous trichiasis; the trachoma local program has provided mass administration of 20 mg/kg of azithromycin in a single dose for children up to 14 years of age or a single dose of 1 g for people aged 15 years and over since 2012. Additionally, the environmental health program has promoted basic sanitation in the communities, and the local government has provided a few sewage and aqueduct systems and latrines, while house-to-house visits during the mass administration of azithromycin have provided education on facial hygiene; however, the population’s adherence to the “F” component of the strategy remains unclear, and education sessions once a year would be insufficient for behavior change.

This study explored the knowledge, beliefs, and practices around the “F” component of the SAFE strategy within the indigenous communities affected by trachoma in Vaupés.

## 2. Materials and Methods

We used a mixed approach to understand the behavior changes in this intercultural context [12] that included two types of observational descriptive studies to investigate aspects around the origin, prevention, transmission, and treatment of trachoma from the indigenous perspective, identifying the knowledge and beliefs about hygiene and cleanliness, their relationship with this disease, and the attitudes and practices regarding the exposure to risk factors. The types of studies developed were the following: The quantitative component of the study was a survey of the knowledge, attitudes, and practices (KAP) related to trachoma, while the qualitative component included two focus-group discussions—from here on, referred to as “focus groups”—one for men and one for women.

To develop the KAP survey tool, we searched for tools in the scientific literature on trachoma in an intercultural context. We found tools for other infectious diseases that we adapted for trachoma [13,14,15]. We grouped the questions by theme into seven modules: The 13 questions in the first module included basic demographic information, the role of the participants at home and in the community, and the risk factors for trachoma; the second module had 17 questions about the knowledge of the origin and transmission of trachoma; the third module included 9 questions about attitudes toward the risk or protective factors for trachoma; the fourth asked 12 questions about the practices related to the prevention and treatment of trachoma; and the fifth module had 2 questions on communication media use for information on health-related issues. Two additional modules looked for information on trichiasis surgery and antibiotic ointment acceptance and adherence, but their analysis was not part of this study.

A health anthropologist, a social worker, a physician, and a nurse validated the data-collection tool in an indigenous community in Mitú; this consisted of identifying whether the language used was understandable and the response options were clear, estimating the time to complete the survey, and identifying the need for new questions.

We used the 2013 trachoma census database of households used for azithromycin mass administration as a sampling frame of the KAP survey; it included 3031 households distributed in 220 communities, the total of the rural area of Vaupés. The sample size was calculated using the statistical package IBM SPSS Statistics version 24, IBM Corporation, New York, NY, USA, with an expected frequency of 50% and a margin of error of 5%; in total, 341 households were required. We selected households through simple random sampling in the azithromycin mass administration database of the rural communities in Vaupés.

The interviewers had instructions to alternate between male and female participants to obtain an equitable representation of each. The study inclusion criteria were being indigenous or mestizo, the male or female head of the household, over 15 years of age, having at least one child, living one or more years in the community, and giving his/her informed consent in writing to participate in the study. If the selected head of household was not present, the closest head of household in the same community who met the inclusion criteria was included.

To perform the KAP survey, we obtained written consent of the Vaupés Regional Indigenous Council (CRIVA), verbal consent of the captains (leaders) of each community, and written consent of each respondent, after explaining the objectives, risks, and benefits of the survey participation and data confidentiality. The group of interviewers was made up of 20 trachoma program technicians, most of whom were previously trained indigenous bilingual health workers. An average time of 2 h was estimated to complete each survey.

During the field work, a local language translator was used if necessary. The surveys were written on paper and then transcribed into Excel by clerks hired for this purpose.

The principal investigator checked the consistency and completeness of all survey forms; the inconsistencies were resolved by verifying the hard copies.

We used IBM SPSS Statistics software to calculate the simple and relative frequencies for each qualitative variable disaggregated by sex. Missing answers were categorized as “Did not answer” and were excluded from the denominator to calculate the relative frequencies. The Fisher exact test was used to identify significant differences between men and women, when 20% or more expected values of the contingency table were less than 5; otherwise, we used *X*^2^.

The KAP survey was conducted between 2014 and 2015, during the third and fourth rounds of the mass administration of azithromycin carried out in the rural communities in Vaupés.

We developed the qualitative component by using the focus-group discussion technique and prepared the following list of questions and counter-questions on the knowledge requirements around five aspects, which were to identify the knowledge about the prevention, treatment, and origin of trachoma; to identify knowledge and beliefs around hygiene and cleanliness; and to identify other topics outside the focus of this study. Some of the questions asked were the following: Is this eye normal? What is this disease called in your language? Why does this happen to people? What does cleanliness mean to you? How do you keep children clean? Is it okay for children to have nasal and eye secretions? Have you heard of trachoma in your community? How do you think trachoma can be prevented? What media do you listen to in the community? In addition to the selected questions, we used discussion-generating questions alluding to trachoma and counter-questions to focus the discussion, as well as showed photos of healthy and trachoma-affected eyes to identify normal and abnormal eyes and what would cause these abnormalities. Then we used mirrors for self-assessment of the participants, allowing them to qualify their own condition. This tool is available in the Supplementary Materials.

We organized two focus groups, one composed of 13 men and one of 8 women, to avoid a passive role of women during the sessions associated with a patriarchal culture in the indigenous population; they were all adults. The number of focus groups and the minimum and maximum number of participants in each group complied with the existing standards suggested for homogeneous populations, looking for the representativeness and information saturation described in the literature [12,16,17]. The participants in the focus groups were selected through purposive sampling by the key informant method [18], guaranteeing the participation of men, women, and healthy and sick people, selected among the group of patients, translators, and companions from rural communities coming to the ophthalmological brigade convened by the Ministry of Health in Mitú. The selection criteria were being an adult, having received care from the trachoma program in the community of residence, and being indigenous; they belonged to similar rural communities, with a similar socioeconomical status.

A public-health anthropologist with experience in qualitative research and work in ethnic communities moderated the focus groups; two observers recorded the sessions and wrote notes on the nonverbal language of the participants; a photographer took photos; and, when necessary, two bilingual interpreters mediated the dialog between Spanish, Tukano, and Cubeo, the dominant languages in Vaupés. The dialog sessions of the focus groups were approximately 4 h each. The criteria for finalizing the sessions were when the new responses in each focus group no longer contributed new information.

The anthropologist manually extracted the information from the recording and field notes into a double-entry matrix. The testimonies were organized according to the deductive and inductive categories generated from the questions and responses [19].

We used the term “ancestral medicine” when the participants used ancestral resources such as prayers, curses, payments, and energy balance, and we used the term of “non-ancestral medicine” for allopathic health resources such as hygiene, flies on the face, nasal secretions, conjunctivitis, and soap.

The analysis of the information included a methodological triangulation from the KAP survey, focus groups, and a review of the literature for confirmation of the findings and to increase the validity [12,20,21,22].

All types of studies had the written consent of the participants. The Ethics Committee of the Higher School of Ophthalmology of the Barraquer Institute of America approved the study protocol through Act 007 of 2014.

## 3. Results

The sociodemographic characteristics of the participants in both studies were as follows: in the KAP survey, 357 people were surveyed, and in the two focus groups, addressing men and women separately, 21 people were interviewed, all over 18 years of age. The role of the participants at home and in the community was evaluated only for the KAP survey; participants in the focus groups were patients with trachomatous trichiasis, companions, or translators (Table 1).

### 3.1. Origin and Transmission of Trachoma

The KAP survey revealed a misconception of the transmission of the disease, associating it with dirty water, river water, and contaminated food and air, but when we added the response options described in the literature (lack of cleanliness of the face and eyes, passes from one person to another, and flies), we observed 39.9% of hits; furthermore, 19% of the answers included poor bodily hygiene and the presence of garbage. We did not find statistically significant differences between men and women.

The most frequent response concerning cleanliness was bathing one or more times a day (67.2%), which was statistically significantly higher in women; in total, 21.6% of the surveyed people associated cleanliness with the use of commercial soap, with an equal proportion in both sexes. Furthermore, 5.9% associated it with resources of the ancestral medicine (prayers) with a greater proportion of men, and this difference was statistically significant; a similar percentage associated cleanliness with ancestral methods to obtain soap from plants.

A total of 40% of the respondents identified taking medication as a trachoma treatment method; 32.8% believed that they would follow the recommendations of ancestral medicine, especially men; and 20.7% associated prevention with good personal hygiene (Table 2).

The focus groups identified traditional causes of trachoma as evil and envy, consumption of jungle animals at certain times of the year according to their ecological calendar, a lack of prayer, and a lack of protection of food. Some testimonials from the participants were the following:

*“There are several people who do not care for our culture by eating mojojoy (Rhyncophorus palmarum beetle larva) from the pupuña palm (Bactris gasipaes) despite knowing that it produces trachoma disease.”*

“According to the *Payé,* conjunctivitis and toothache, appear during the time of the *chontaduro* and *tapurú”* (caterpillars of butterflies of the genus *Automeris*).

The participants also stated that a lack of prayer and a lack of the protection of food is the cause of trachoma:

*“To prevent trachoma, you must cover the food, wash your hands, cover the pots.”*

*“If families pray for protecting their children, they won’t get diseases like trachoma.”*

The participants recognized the importance of using both ancestral and non-ancestral medicine (scientific medicine):

*“Ancestral medicine does not cure trachoma, when the disease starts during childhood, causes blindness; sometimes non-ancestral doctors cure the disease but in other cases they don’t.”*

*“Non-ancestral doctors give education and cure people when ancestral medicine doesn’t work.”*

### 3.2. Attitudes toward Trachoma Prevention in the Community

The male participants in the KAP survey prioritized informing the non-ancestral doctor about the occurrence of trachoma in the community, while women prioritized receiving the medical brigade; in both cases, the difference was statistically significant. The participants identified keeping the faces of children, young people, and adults clean to prevent trachoma, but they did not identify teaching other people about it; taking medication, reporting to the ancestral doctor (*Payé* or *Cumú*), and maintaining hygiene and cleanliness measures were the other aspects mentioned (Table 3).

The attitudes toward the prevention of trachoma, narrated by the participants of the focus groups, revealed that the adoption of preventive measures has a variable impact between individuals and families; similarly, it was identified that practices such as the development and use of plants to make soap are becoming extinct because commercial soap is preferred:

*“When you give hygiene education, some families accept it, others don’t; it’s very difficult.”*

*“Some people clean their children, others don’t; they learn little by little.”*

Facial cleaning in children to prevent trachoma is the responsibility of women in the communities, according to the male comments:

*“The health workers educate on hygiene, cleaning the home’ some families do it, especially women do not pay attention, some reject education.”*

*“The use of plant soap and foams has been lost because now commercial soap would be available.”*

### 3.3. Hygiene Practices

The participants in the KAP survey identified the importance of washing children’s faces many times a day, even if they seem healthy (61.9%), and of increasing the frequency when having nasal and/or conjunctival discharge (91.3%). However, the remaining risky practices included sharing towels among family members after bathing (52.7%) and cleaning secretions from the nose and eyes with a rag towel or clothing in use (66.1%) or with hands and fingers—the latter higher in men, with statistically significant differences (Table 4).

In all, 40% of the KAP survey participants preferred health messages to be broadcast through the radiotelephone network, 33.9% preferred education visits house-to-house, and 8% preferred through the radio.

Women participating in the focus groups recognized that carelessness in the hygiene of their children is a risk factor for trachoma:

*“The lack of care from the mothers leads to secretions sticking to the eyelashes and become infected by that disease.”*

*“The mother did not bathe the child, causing trachoma and other transmissible diseases.”*

The participants in the focus groups recognize that the elderly use plant leaves as part of their body hygiene ritual, but younger people no longer use it:

*“We, indigenous people, wash our faces with water, and older people combined it with crushed leaves from the forest.”*

The participants mentioned that at the age of seven, children already know how to clean themselves, as well as the role of sisters in the care of other children:

*“When children are 7 years old, they already know how to wash their faces. The sisters are the ones who take care of the children; they are the ones who wash the hands, the face.”*

The focus-group participants identified that ancestral medicine practices are maintained to prevent trachoma:

*“My husband protects our children and grandchildren through prayers against serious diseases; by doing that they protect families and children from diseases like trachoma.”*

## 4. Discussion

Knowledge of, attitudes toward, and practices for the prevention of trachoma using ancestral medicine persist in the indigenous communities of Vaupés; some of them coincide in their methods of prevention with those of scientific medicine, especially in relation to the need to maintain good hygiene and health. Those that do not coincide can be classified into two groups: those of ancestral medicine (prayers, diets, curses, and the use of medicinal plants) and those that correspond to habits (sharing towels and cleaning children’s nasal secretions with clothing or hands) due to economic limitations or due to ignorance of their implications in visual health; both categories can be addressed with intercultural educational strategies sustained over time.

### 4.1. Protective and Risky Practices for Trachoma

Daily regular baths and washing children’s faces with water for conjunctivitis are protective factors for trachoma; however, cleaning children’s nose and eyes with hands or sharing towels or clothes in use and not having permanent access to commercial or traditional soaps contribute to its transmission. Washing faces with water and soap “as frequently as necessary”, at least every 4 h, is more effective at removing ocular discharge than just using water or wiping with a hand; it contributes to removing the contaminated secretion and avoids transmission [23]. In the Amazon region, having access to rainwater all year round does not require a large-scale investment as in other regions of the world [24]; however, despite the acceptance of commercial soap for face washing and laundry, it is not regularly available and is expensive, while producing traditional and herbal foams and soaps is not currently a frequent practice.

Educational messages emphasizing the daily use of ancestral or commercial soap in facial hygiene routines, using clean individual towels to dry the face and body after bathing, discouraging the removal of nasal and eye secretions with the hands and clothing, and prioritizing soap-washing of the clothing that is most likely to come into contact with nasal or eye secretions are a priority [25], in addition to managing the provision of soap for these populations. Community behavior-change programs must provide resources such as soap and require long intervention throughout the year [25]. The SAFE strategy should combine washing of clothes, cleanliness in the home, and facial cleanliness in educational campaigns, as general cleanliness facilitates the adoption of the “F” component [26]. A program of education and provision of enablers such as soap and water for school students is not enough to achieve a change in behavior throughout the community; this suggests the need for long-term programs that involve all age groups [27].

Health authorities can promote hygienic habits and the mass administration of azithromycin through community-accepted media such as radiotelephone networks, home visits, and radio broadcasts.

### 4.2. The Role of Women

In the KAP survey, the participation of men was predominant; although in the survey protocol, the interviewers were instructed to alternate the participation of men and women, in most households, it was the man who answered the questions. The lower representation of women was associated with the dominant role played by men at home; triangulation of information, combining quantitative and qualitative methods, was helpful in understanding the women’s perspectives [20,21].

Given that women spend more time at home than men and take care of their children, as in most cultures [28], we consider that the educational strategy should be based on volunteer women and mothers in their role as promoters of healthy habits and social development in endemic communities. Women and healthcare teams should work together in a network to conduct community visits, exchanging experiences of best practices with the support of community leaders and local health authorities [29]. Likewise, indigenous girls take care of the hygiene of the youngest children, so educational campaigns should include them through an age-appropriate strategy designed to enable them to be health promoters of behavior change. Health teams can distribute azithromycin and monitor the operation and functioning of the community network.

### 4.3. The Need to Advance the Sociocultural Adaptation of the “F” Component of the SAFE Strategy

We recognize the need to understand and honor the knowledge of indigenous ancestral medicine and the social and cultural aspects that support it in order to offer effective and sustainable educational programs [30]. The implementation of the “F” component of the SAFE strategy should be minimally intrusive to the traditional lifestyles of the indigenous people. The holistic understanding of the phenomenon of health and disease in this population makes the introduction of new and foreign habits into their culture take longer and requires an intercultural approach to promote them by properly trained health workers [11,31,32,33,34].

The sociocultural adaptation of this component of SAFE must start from the recognition of the existence of an ancestral health system that has epistemological bases, agents, and resources different from non-ancestral medicine. The intercultural approach should use legitimate spaces and moments with indigenous authorities for the dialogue to integrate the knowledge of both medicines and equal conditions.

By law, the Colombian State preserves the ancestral medical system and considers it a cultural heritage of the nation [35]. The indigenous authorities demand compliance with the legal framework for negative impact on their health, culture, and survival associated with non-ancestral medicine interventions; this makes it necessary to establish consultation and dialog between health authorities and indigenous leaders, as established in the Law 21 of 1991 of Colombia.

Our results revealed the community recognition of the limitations of ancestral medicine to prevent and treat trachoma and the need to combine both medical systems to improve the sustainability and impact of interventions. Applying qualitative research methods to recognize and understand indigenous communities’ knowledge, attitudes, and practices is crucial for the appropriate design and implementation of intercultural health programs in Vaupés and other Amazonian departments.

The limitations of our study include that we did not attend to differences in the responses associated with age in either of the methodologies implemented, because age was excluded from the data-collection tools. Likewise, we acknowledge that the responses of the participants mediated by a translator from Tukano or Cubeo to Spanish may have had a certain degree of distortion.

## 5. Conclusions

This study explored the knowledge, beliefs, and practices of indigenous people in Vaupés toward the SAFE strategy for preventing trachoma versus their ancestral beliefs and practices. In Vaupés, four years after the implementation of the SAFE strategy, a lack of knowledge and risky practices for trachoma transmission persist in the indigenous population. The “F” component of the SAFE strategy was implemented in annual education and promotion rounds, together with the mass administration of azithromycin, but this may be insufficient for changes in attitudes and practices.

The indigenous community’s adoption of the “F” component is prevented by their limited knowledge of the origin and transmission of trachoma and cleanliness concepts.

The introduction or strengthening of new habits, such as general hygiene, facial cleaning, and washing clothes with soap to prevent trachoma, requires an intercultural approach, financial support, continuous community education more than annually, intercultural training for health workers and leaders of the same community, and the provision of commercial soap. Indigenous parents and schoolteachers play a crucial role in the care and hygiene at home, as well as girls, who care of their younger siblings, which must be empowered to be effective and sustainable.

Qualitative studies on the knowledge, beliefs, and practices of indigenous population on the “F” component of the SAFE strategy are necessary for the correct design and implementation of the intercultural approach to develop competences in the day-to-day life, to prevent trachoma.

Integrating ancestral and non-ancestral knowledge in the implementation of the “F” component of the SAFE strategy facilitates the participation and engagement of the indigenous stakeholders to integrate the strategy in many aspects of the daily life of the community; this is especially important in zones with no permanent health workers.

## Figures and Tables

**Table 1:** The sociodemographic characteristics and the roles of the population studied in terms of the trachoma knowledge, attitudes, and practices survey and focus groups.

Aspects Evaluated		KAP Survey		Focus Groups ^5^	
		*n* ^1^	%	*n* ^1^	%
Participants over 18 years old		357	100	21	100
Participating indigenous		357	100	21	100
Participating men		260	72.8	13	61.9
Participating women		97	27.2	8	38.1
Ethnicities represented ^2^		29	69.0	14	33.3
Communities represented ^3^		160	72.7	14	6.4
Sedentary settlement pattern		334	93.6	19	90.5
Roles within the family	Mother	88	24.6	-	-
	Father	208	58.3	-	-
	Other	61	17.1	-	-
Role in the community ^4^	*Payé-Cumú*	61	17.1	-	-
	Community leader	125	35.0	-	-
	Teacher	28	7.8	-	-
	Health promoter	25	7.0	-	-
	No leadership role	155	43.4	-	-

^1^ The value corresponds to the total number of participants. ^2^ The denominator of the rural indigenous ethnic groups is 42, source [6]. ^3^ The denominator of the indigenous communities (villages) is 220, source [6]. ^4^ Multiple answers are possible. ^5^ The focus groups are TT patients, their companions, or translators.

**Table 2:** Knowledge about the origin, transmission, prevention, and treatment of trachoma, along with cleanliness, according to sex, in the knowledge, attitudes, and practices survey.

Aspects Evaluated	Sex of Respondent						*p* ^4^
	Male *n* = 260		Female *n* = 97		Total *n* = 357		
	*n* ^1^	% ^2^	*n* ^1^	% ^2^	*n* ^1^	% ^2^	
**Origin and transmission of trachoma**	**393**		**126**		**519**		
Other (dirty water, contaminated food or air, river water, and other)	80	30.8	22	22.7	102	28.6	0.132
Lack of cleanliness in the face and eyes ^3^	77	29.6	24	24.7	101	28.3	0.462
Did not know	61	23.5	24	24.7	85	23.8	0.670
Due to a lack of cleanliness ^3^ (body)	47	18.1	13	13.4	60	16.8	0.353
Passes from one person to another ^3^	40	15.4	16	16.5	56	15.7	0.697
Due to flies ^3^	39	15.0	11	11.3	50	14.0	0.438
Due to garbage ^3^	32	12.3	8	8.2	40	11.2	0.323
Did not answer	10	3.8	7	7.2	17	4.8	0.183
Ancient resource elements (spirit and evil) ^3^	7	2.7	1	1.0	8	2.2	0.345
**The cleanliness concept is associated with the following:** ^3^	**316**		**109**		**425**		
Bathing one or more times per day	171	65.8	69	71.1	240	67.2	0.462
Use of commercial soap	56	21.5	21	21.6	77	21.6	0.937
Other	31	11.9	11	11.3	42	11.8	0.824
Did not answer	19	7.3	5	5.2	24	6.7	**-**
Ancestral methods for obtaining soap from plants	19	7.3	2	2.1	21	5.9	0.055
With prayers or ancestral medicine	20	7.7	1	1.0	21	5.9	**0.015**
**Trachoma prevention and treatment**	**386**		**115**		**501**		
Taking medicine ^3^	108	41.5	35	36.1	143	40.1	0.525
Following the recommendations of ancestral medicine ^3^	94	36.2	23	23.7	117	32.8	**0.045**
Consulting health workers (non-ancestral) ^3^	70	26.9	17	17.5	87	24.4	0.099
With good personal hygiene measures ^3^	58	22.3	16	16.5	74	20.7	0.306
Did not know	38	14.6	14	14.4	52	14.6	0.905
Did not answer	10	3.8	8	8.2	18	5.0	-
Other	6	2.3	2	2.1	8	2.2	0.935
Cannot be prevented	1	0.4	0	0.0	1	0.3	-
It does not produce anything	1	0.4	0	0.0	1	0.3	-

^1^ Number of responses associated with each aspect evaluated. ^2^ Proportion with respect to the total number of men or women surveyed. ^3^ Multiple answers are possible. ^4^ Statistical significance using the chi-square test. Subheadings in bold correspond to a category that groups the subsequent aspects evaluated.

**Table 3:** Participant responses (by sex) to their contribution to prevent trachoma in the community in the knowledge, attitudes, and practice survey.

Aspects Evaluated	Sex of the Respondent						*p* ^3^
	Male *n* = 260		Female *n* = 97		Total *n* = 357		
	*n* ^1^	% ^2^	*n* ^1^	% ^2^	*n* ^1^	% ^2^	
**How would you help the community? ^4^**	**481**		**169**		**650**		
Informing the health workers (non-ancestral)	126	48.5	36	37.1	162	45.4	**0.036**
Keeping the faces of children, young people, and adults clean	116	44.6	36	37.1	152	42.6	0.150
Taking medication	77	29.6	27	27.8	104	29.1	0.660
Informing the *Cumú/Payé*	36	13.8	14	14.4	50	14.0	0.941
Keeping the home clean to avoid flies	32	12.3	11	11.3	43	12.0	0.755
Accepting the medical brigade	23	8.8	16	16.5	39	10.9	**0.046**
Participating in workshops or meetings on trachoma	24	9.2	12	12.4	36	10.1	0.413
Collaborating in the care of trichiasis-operated patients	20	7.7	8	8.2	28	7.8	0.901
Keeping towels, rags, and toiletries clean	14	5.4	6	6.2	20	5.6	0.802
Did not answer	13	5.0	3	3.1	16	4.5	-
Teaching others the importance of washing the face	0	0.0	0	0.0	0	0.0	-

^1^ Number of responses associated with each aspect evaluated. ^2^ Proportion with respect to the total number of men or women surveyed. ^3^ Statistical significance using the chi-square test. ^4^ Multiple answers are possible. Subheadings in bold correspond to a category that groups the subsequent aspects evaluated.

**Table 4:** Hygiene practices for adults and children according to sex in the trachoma knowledge, attitude, and practice survey.

Hygiene Practices	Sex of the Respondent						*p* ^3^
	Male *n* = 260		Female *n* = 97		Total *n* = 357		
	*n* ^1^	% ^2^	*n* ^1^	% ^2^	*n* ^1^	% ^2^	
**Sharing clothes to dry the body after bathing**							
Yes	142	54.6	46	47.4	188	52.7	0.226
No	118	45.4	51	52.6	169	47.3	
**Daily frequency of washing the face of healthy children**							
Many times	154	59.2	67	69.1	221	61.9	0.116
Few times	86	33.1	21	21.6	107	30.0	
Never	7	2.7	2	2.1	9	2.5	
No answer	13	5.0	7	7.2	20	5.6	
**Daily frequency of washing the face of children when they have nasal or conjunctival discharge**							
Many times	238	91.5	88	90.7	326	91.3	0.931 ^4^
Few times	11	4.2	3	3.1	14	3.9	
Never	7	2.7	2	2.1	9	2.5	
No answer	4	1.5	4	4.1	8	2.2	
**Elements for cleaning children’s nose and eyes ^5^**							
With a rag towel or clothing in use	173	66.5	63	64.9	236	66.1	0.965
With water	127	48.8	48	49.5	175	49.0	0.828
With hands or fingers	36	13.8	5	5.2	41	11.5	**0.023**
No answer	11	4.2	8	8.2	19	5.3	
Other	8	3.1	4	4.1	12	3.4	0.610

^1^ Number of responses associated with each aspect evaluated. ^2^ Proportion with respect to the total number of men or women surveyed. ^3^ Statistical significance using chi-square test. ^4^ Statistical significance using the Fisher exact test. ^5^ Multiple answers are possible. Subheadings in bold correspond to a category that groups the subsequent aspects evaluated.

## Data Availability

The data presented in this study are available upon request from the corresponding author. The survey form is also available in the Supplementary Materials.

## Supplementary Materials

The following supporting information can be downloaded at: https://www.mdpi.com/article/10.3390/ijerph20054632/s1. KAP questionnaire, guiding instrument for conducting focus groups, approval act of the research ethics committee (Act 007 of 2014), educational material (drawings), and an intercultural approach and pedagogical proposal to include trachoma education in primary schools.
