# Supplementary material for: Knowledge, Attitudes, and Practices of Hygiene and the Prevention of Trachoma in the Indigenous Population of the Colombian Amazon Vaupés Department
Source: Int J Environ Res Public Health. 2023 Mar 6;20(5):4632. doi: 10.3390/ijerph20054632 (PMC10001660; doi:10.3390/ijerph20054632)
Supplement: Supplementary file 1 [file ijerph-20-04632-s001.zip › ijerph-2146773-supplementary.pdf]

Supplementary Material

SURVEY OF KNOWLEDGE, ATTITUDES AND PRACTICES ABOUT TRACHOMA

The Ministry of Health and Social Protection (MSPS), in agreement with the ESE Hospital San Antonio de Mitú and with the collaboration of the Research Department of the Higher School of Ophthalmology of the Barraquer Institute of America conduct a survey in your community, the intention is asking you some questions and get information to improve the results of the National Neglected Infectious Diseases Program.

Your participation is completely voluntary. All information you provide will remain confidential and your name will not be used in any published report of this survey. We appreciate the time provided and your contribution to improving the information, education and communication strategies of the Department of Vaupés and the Nation.

SECTION 1. GENERAL DATA

1. General data: Number of Survey: [__] **Date:** Day [__] Month [__] Year [__] **Start time:** [_______]

2. Name of the respondent or respondent: ____

3. Requires translator: 1. Yes [__] 2. No [__] In which language: ____________________________

4. How many people live in this house? [___]

5. How many children under the age of 10 live with you: [___] (If the answer is that there are no children in the house, the survey is not applied. Kindly explain that you are not a candidate and why you are not and that this fact will not affect you regarding the benefits offered by the project)

6. Ethnic group or indigenous people to which it belongs: ___________________________________

7. Settlement pattern:

1. [__] Lives all the time in the same place (sedentary)
2. [__] You move your place of residence to other places frequently but return to a base community (semi nomad)
3. [__] Moves to a permanent place of residence, does not have a base community (nomadic)

8. Place of residence of the respondent: zonal (write zone code)

1. [___/___] 2. Name of the community: ____

9. Sex of the respondent: 1. [__] Male 2. **[__]** Female

10. Role of the respondent (The main role or roles that the respondent plays in the community are selected, several can be chosen)

In the family:

1. [__] Mother 2. [__] Father 3. [__] Other

In the community:

1. [__] Ancestral doctor / Payé 2. [__] Cumú 3. [__] Community leader 4. [__] Teacher
5. [__] Health Agent 4. [__] Teacher 6. [__] Other. Which? _________________

11. Can you read and write? (If the answer is No, go to question 11) 1. [__] Yes 2. No [__]

Respondent's level of education:

1. [__] None 2. [__] Completed primary school 3. [__] Incomplete primary school
4. [__] Complete high school 5. [__] Incomplete secondary school 6. [__] Other. Which? ______________

12. How many people between the ages of 6 and 18 attend school? [____]

13. Where do you get your water from?

1. [__] River/stream/Small river 2. [__] Rainwater 3. [__] Aqueduct 4. [__] Other

14. What do you use the water for? (Identify the two most important, the main one with the number 1 and high school with number 2) (Ask the question, but don't read the answer options; select the options that best fit).

1. [__] Prepare food 2. [__] Personal hygiene 3.[__] House cleaning
4. [__] Do the laundry 5. [__] Spiritual rituals 6. [__] Other

SECTION 2. KNOWLEDGE OF THE DISEASE

15. How often do the children in your house have pus or crusty eyes or conjunctivitis? (Ask the question, but don't read the answer options; select an option).

1. Many times [__] 2. Rarely [__] 3. [__] Sometimes 4. [__] Never 5. [__] Don't know.

16. Do you know if flies that land on people's faces cause health problems? (Ask the question, but don't read the options response; select the options that best fit)

1. [__] Don't know(go to the next question) 2. [__] Conjunctivitis 3. [__] Skin diseases
4. [__] Red eyes 5. [__] Diarrhea 6. [__] They chase the children.
7. [__] Not good(does not identify any of the above) 8. [__] Produces nothing 9. [__] Others

17. Do you know what people in your family do when flies land on their faces? (Ask the question, but don't read the options answer; select the options that best fit)

1. [__] They scare them away 2. [__] Does nothing
3. [__] Doesn't notice when flies land on his face. 4. [__] Don't know

18. Do you know how discomfort in the eyes (crud, pus, conjunctivitis) is produced? (Do not read the answer, you can select more than one option)

1. [__] Don't know 2. [__] Because of the dirty water
3. [__] By Air 4. [__] passes from one person to another.
5. [__] Due to lack of cleanliness of the face and eyes 6. [__] Due to lack of cleanliness
7. [__] For contaminated food 8. [__] For the garbage
9. [__] Because of the flies 10. [__] By the water of the river, pipe
11. [__] By an evil spirit 12. [__] For evil
13. [__] Does not respond 14. [__] Other: ____________________

19. Do you know how to prevent and cure conjunctivitis or rheum or pus in the eyes? (Ask the question, but don't read the answer options; select the options that best fit)

1. [__] Don't know 2. [__] Taking medication (drops, creams)
3. [__] Following the recommendations of ancient medicine 4. [__] can't be prevented.
5. [__] Consulting the health worker(not ancestral) 6. [__] With good personal hygiene measures
7. [__] Other: ______________________ 8. [__] Doesn't produce anything.

20. Do you know what is the name in your language of the disease that causes conjunctivitis or pus or rheum in the eyes?

1. [__] If you know. Which? _____________________ 2. [__] Don't know 3. [__] Does not respond.

21. Do you know the name of the disease in medicine (not ancestral) that causes conjunctivitis or pus or discharges in the eyes? (if the answer is trachoma, indicate that you do know, but do not prompt a response)

1. [__] If you know. Which?________________________ 2. [__] Don't know 3. [__] Does not respond.

22. Point out who told you the name of the disease that causes conjunctivitis or pus or discharges in the eyes:

1. [__] Don't know 2. [__] Health promoter (non-ancestral) 3. [__] Ancient Physician
4. [__] Cumú 5. [__] Neighbor 6. [__] Family
7. [__] Medical (non-ancestral) 8. [__] Community leader 9. [__] Others. Who?__________

23. Have you observed or seen in your community people who present any of the following aspects? (Read each of the following options and wait for the answer)

1. [__] I have not seen 2. [__] No tabs
3. [__] Dull eyes 4. [__] That the eyelashes are removed.
5. [__] Droopy eyelids 6. [__] That they are blind.
7. [__] Eyelashes that go into the eyes 8. [__] People who have undergone eyelid surgery.
9. [__] Does not respond

24. Based on the previous answer, do you know if some people in your community have eye problems?

1. [__] There is none 2. [__] Youth 3. [__] Adults 4. [__] Elders

25. Do you know how the disease that makes the eye opaque – white, droopy eyelids, eyelashes that get into the eyes, no eyelashes, eyelashes removed, blindness, eyelid or eye surgery? (Do not read the answer, you can select more of one option)

1. [__] Don't know 2. [__] For eyelashes that are buried in the eyes
3. [__] For the Water 4. [__] By Air
5. [__] passes from one person to another 6. [__] Due to lack of cleanliness of the face and eyes
7. [__] Due to a blow or trauma 8. [__] By insect bite
9. [__] It's normal because of old age 10. [__] A fleshiness that grows inside the eye.
11. [__] By an evil spirit 12. [__] For an evil
13. [__] Does not respond 14. [__] Other: ______________________________

26. Do you know or don´t you know the name in your language of the disease that causes the eyelashes to bury themselves in the eye?

1. [__] If you know. Which?________________ 2. [__] Don't know 3. [__] Does not respond.

27. Do you know, or do you know the name in medicine (non-ancestral) of the disease that causes the eyelashes to bury themselves in the eye? (if the answer is trachomatous trichiasis, indicate if knows, but do not induce an answer)

1. [__] If you know. Which? _______________ 2. [__] Don't know 3. [__] Does not respond.

28. Point out who told you the name of the disease that causes the eyelashes to bury themselves in the eye:

1. [__] Don't know 2. [__] Health agent 3. [__] Cumú / Payé 4. [__] Neighbor
5. [__] Family 6. [__] Doctor (not ancestral) 7. [__] Some community leader 8. [__] Other

29. Do you know or don´t you know if the eyelashes that are buried in the eye can leave the person blind?

1. [__] Yes you know 2. [__] Don't know 3. [__] Does not respond.

30. How is vision loss prevented when the eyelashes are buried in the eye? (Ask the question, but don't read the answer options; select the options that best fit)

1. [__] Don't know 2. [__] Taking medication (drops, creams)

3. [__] Following the recommendations of ancient medicine
4. [__] It can't be prevented.
5. [__] Consulting the health worker (non-ancestral)
6. [__] With good personal hygiene measures
7. [__] Taking off the eyelashes
8. [__] With surgery
9. [__] Other. Which?_________________________
10. [__] Does not respond.

31. Have you heard one or more of the following words? (Read each answer option and mark the one that corresponds)

1. [__] trachoma
2. [__] Trichiasis
3. [__] Conjunctivitis
4. [__] Azithromycin
5. [__] Tetracycline
6. [__] I haven't heard any.

SECTION 3. ATTITUDES

32. Do you have the habit of cleaning the face and eyes of healthy children during the day?

1. [__] Very frequent (3 or more times)
2. [__] Infrequent (once or twice)
3. [__] Never

33. How often do children clean their faces and eyes when they have conjunctivitis or pus or crusts?

1. [__] Very frequent (3 or more times)
2. [__] Infrequent (once or twice)
3. [__] Never

34. What would you do if you or someone in your family buried their eyelashes in their eyes? (Don't read him the answer)

1. [__] They consult the health worker (non-ancestral)
2. [__] They consult the ancestral doctor (Payé, Cumú)
3. [__] Buried eyelashes are removed
4. [__] Home remedies are made (plants, prayers, etc.)
5. [__] They do nothing
6. [__] Others. Which? ________________________

35. If the solution to cure trichiasis was to have eyelid surgery, would you do it?

1. [__] Yes
2. [__] No
3. [__] Don't know
4. [__] Does not respond.

36. Why wouldn't you operate in Mitú? (Select one or more of the following possible options that best fit the answer)

1. [__] For fear of pain
2. [__] Due to negative comments from other people who underwent surgery
3. [__] For beliefs that do not allow it
4. [__] They don't think it's necessary
5. [__] There is mistrust in hospitals, EPS (insurance company)
6. [__] For having to leave his family unprotected for a long time
7. [__] Because of the geographical difficulty
8. [__] Out of fear of the unknown
9. [__] Others. Which? ____

37. Do you agree that the auxiliary, promoter or medical brigade visit your community to carry out actions to treat these diseases? (Specify, why not?)

1. [__] Yes
2. [__] No
3. [__] Don't know
4. [__] Does not respond.

Why not? ____

38. Do you agree that the auxiliary, promoter or medical brigade give each of the members of the community one or two antibiotic pills to treat these diseases? (Specify, why not?)

1. [__] Yes
2. [__] No
3. [__] Don't know
4. [__] Does not respond.

Why not? ____

39. Could you help the community avoid blindness from trachoma?

1. [__] Yes 2. [__] No 3. [__] Don't know 4. [__] Does not respond.

40. How could you help these people in your community? (Ask the question, but don't read the answer options; select the options that best fit)

1. [__] Teaching and remembering to wash the face, remove mucus, rheum, and pus in the eyes in children and adults
2. [__] Teaching and reminding to take the medicines
3. [__] Informing the health worker (non-ancestral)
4. [__] Informing the Cumú/ Payé
5. [__] Collaborating in the care of the person when they do the surgery
6. [__] Teaching and reminding to keep the house clean to avoid the presence of flies
7. [__] Teaching and remembering to keep towels, rags, and clothes used for cleaning clean
8. [__] Participating in workshops or meetings that discuss the topic
9. [__] Receiving the medical brigade

SECTION 4. PRACTICES

41. What is it to be a clean person? (Do not read the options, accept multiple answers)

1. [__] Associates cleaning with bathing one or more times a day
2. [__] Relate cleaning to ancestral methods to obtain soap from plants
3. [__] Associate cleaning with prayers or ancestral medicine
4. [__] Associates cleaning with the use of soap
5. [__] Others. Which? ____

42. Why be clean? (Don't read the options)

1. [__] To be healthy 2. [__] To be happy 3. [__] To look better, prettier
4. [__] To have more energy to work 5. [__] To make friends 6. [__] It doesn't matter
7. [__] Others. Which? ____

43. How often do you clean the children's eyes when they have conjunctivitis, mucus, pus and crusts? (If the answer is very frequent or infrequent, go to the next question 44. If the answer was never, go to the question 43).

1. [__] Very frequent 2. [__] Uncommon 3. [__] Never

44. Why don't you do it? (Ask the question, but don't read the answer options; select the options that best fit)

1. [__] You don't have what (rag, towel or clothes) 2. [__] It is not necessary to clean the child's face.
3. [__] No time to clean his face 4. [__] Doesn't realize.
5. [__] Not important 6. [__] Other. Which? ______________________

45. What do you use to clean the nose and eyes? (Ask the question, but don't read the answer options; select the options that best fit)

1. [__] With water 2. [__] With a rag, towel or clothing 3. [__] With hands or fingers
4. [__] Other. Which? _________ 5. [__] Does not respond

46. How often do you bathe?

1. [__] Once a day 2. [__] Twice day 3. [__] More than twice a day
4. [__] Once a week 5. [__] Once a month 6. [__] Does not respond.

47. In your family, who is responsible for taking care of small children during the day (except babies in arms)? (Do the ask, but don't read the answer options; select the options that best fit)

1. [__] The mother 2. [__] The father 3. [__] Grandparents 4. [__] A minor (brother, cousin)
5. [__] Older brothers 6. [__] Community home 7. [__] The teacher 8. [__] Uncles
9. [__] Councils, captains 10. [__] Nobody

48. How many times a day do children bathe? (Please tick one option)

1. [__] Once a day 2. [__] Twice a day 3. [__] More than twice a day
4. [__] Once a week 5. [__] Once a month 6. [__] Does not respond.

49. Do you use towels, rags or clothes at home to dry yourself after bathing? 1. Yes [__] 2. No [__]

50. Is it customary to share towels, rags or clothes at home? 1. Yes [__] 2. No [__]

51. Do children use towels, rags or clothes after bathing? 1. Yes [__] 2. No [__]

52. What do you do to prevent flies from proliferating in your community? (Ask the question, but don't read the answer options; select the options that best fit)

1. [__] Doesn't know what to do
2. [__] Knows how to control but does nothing. Because? ______________________________
3. [__] Controls it with ancient techniques. Which is it? ______________________________
4. [__] Request fumigation
5. [__] Burying and/or burning the garbage
6. [__] Keeping the house clean
7. [__] Keeping food covered
8. [__] With smoke
9. [__] Others. Which? ______________________________

SECTION 5. MASS MEDIA AVAILABLE

53. Which of the following means of communication are available to you? (Ask the question, and read the answer options that most adjust)

1. [__] Radiophone 2. [__] Radio station 3. [__] Brochures
4. [__] Posters or posters on the walls 5. [__] Visit house to house 6. [__] None
7. [__] Other. Which? ______________________________

54. Of the means of communication available to you, which one do you use the most?

1. [__] Radiophone 2. [__] Radio station 3. [__] Brochures
4. [__] Posters or posters on the walls 5. [__] Visit house to house 6. [__] None
7. [__] Other. Which? ______________________________

SECTION 6. OF PERCEPTION OF THE ACTIONS OF THE VAUPÉS SECRETARIAT OF HEALTH

55. Do you receive care from health officials when you need it (vaccination, public programs, public health problems, and medical care) ? 1. Yes [__] 2. No [__]

56. Has the Vaupés Health Secretariat carried out actions in your community to prevent and cure trachoma?

1. [__] Yes 2. [__] No 3. [__] Don't know 4. [__] Does not respond.

56a. If the answer is Yes, read the answer options and mark the ones that best fit.

1. [__] Visit to the houses 2. [ ] Eye exam 3. [__] Medication delivery
4. [__] Surgical days 5. [ ] Workshops, talks 6. [__] None
7. [__] Other. Which? ____

ADHERENCE TO THE USE OF OXYTETRACYCLINE OINTMENT (Interviewer)

Verify if in the visited community there were children under six months who participated in the medication round of the previous year; If yes, please visit them and their parents or caregivers ask the following:

57. Who applied the eye cream (oxytetracycline ointment) to the child last year?

1. [__] The Mother2. 2. [__] The Father 3. [__] The Professor 4. [__] A younger brother or sister
5. [__] The promoter 6. [__] Nobody 7. [__] Other: ______________________________

59. How many times did you apply the cream to the child? (Do not read the answers, select the most appropriate)

1. [__] Every day twice for six weeks in both eyes 2. [__] Did not apply it 5. [__] Two weeks
3. [__] Just one day 6. [__] Three weeks 4. [__] One week

60. Why did they send that cream to the boy or girl? (Do not read the answers, select the most appropriate)

1. [__] Don't know 2. [__] To take away the conjunctivitis disease.
3. [__] So he doesn't get trachoma 4. [__] Other: ___________________________

61. Where do you have the bottle with the cream (oxytetracycline) for the boy's or girl's eyes? (Check the answer given and select from the following options)

1. [__] It is stored full or almost full 2. [__] It is stored half used 3. [__] It's almost empty.
4. [__] Not at home 5. [__] He doesn't know where he is.

END OF SURVEY

Observations (Write aspects that do not match the responses of the respondent)

____

____

____

Was the survey completed in its entirety? **1.** Yes [__] **2.** No [__]

Time of completion of the application of the survey: [________________]

INTERVIEWER: ___________________________________ **SIGNATURE:** ____________________

GUIDE FOR FOCUS GRUPOS DISCUSSIONS

NEED TO KNOW	GENERATING QUESTION	COUNTERQUESTIONS	
**1. Identify the knowledge about the prevention, treatment and origin of trachoma.**	1.1 *Slide (photo of eye with advanced trachoma)* What do you see in this photo?	**1.1.1** Is this eye normal, or is something different happening?	**1.1.2** Describe what is not normal in this picture
	**1.2** What is this evil called in language?		
	1.3 Do you know people with this disease?	**1.3.1** Do you know people whose vision has become opaque, who has clouded their vision?	**1.3.2** Who do you know who have opaque views in your family or in your community?
	**1.4** Why does this happen to these people?	1.4.1 What do people do for this to happen to them, that their vision becomes cloudy?	1.4.2 Is it because of other people that this happens with vision? Or is it for something one does?
	1.5 How does this evil start in the eyes?	**1.5.1** What happens in the eyes before they are cloudy?	**1.5.2** Can you tell that a person's vision is going to blur?
	1.6 This evil eye affects children, adults, the elderly, women, men, or who?	**1.6.1** In which group of people does this evil eye occur most frequently?	**1.6.2** All people can be attacked equally by this evil of vision?
	1.7 With opaque vision can people still see? What do you feel and what happens to people who have this disease?	**1.7.1** How does the life of people who start this disease change?	
	**1.8** How is this evil eye cured? this bad	**1.8.1** Do you know people who cure vision? How do they do that?	**1.8.2** What would you do if you realize that you are going to get this bad? ....And if they already have it?
**2. Determine possible barriers to acceptance of surgical treatment. (Brainstorm to prioritize the top five reasons limiting acceptance. Use 4-delay methodology to group responses)**	**2.1** Some are here because they are going to be evaluated to find out if anyone needs eye surgery so that their vision does not become opaque and for those who are already like this, so that their eyes do not hurt anymore or because they are accompanying a relative who have this problem; It was the most difficult thing to make the decision to come to Mitú to have surgery?	**2.1.1** Did you have problems coming to Mitú to cure your own vision problem or that of the family member you are accompanying? Which is it?	**2.1.2** For the people who come to Mitú it is easy to come and stay here for a few days? Because?
	**2.2** Why did you come from so far away to participate in this event?	**2.2.1.** What benefits do you hope to have by being here in Mitú to be evaluated by specialist eye doctors and participate in this journey with the hospital?	
	**2.3.** If someone knows a person who has already been operated on for opaque vision, how did that person do?	**2.3.1** Today people who have already been operated on for this disease still feel pain in their eyes? Do they feel better or worse than before the operation?	
**3. Identify the knowledge and beliefs around hygiene and cleanliness**	3.1 *Slide* (photo of children with eye discharge) What do you see in this photo of children?	3.1.1 What attracts attention in these children?	**3.1.2.** If the children remain dirty, as they are in the photo, can that affect them in any way?
3.2.1	3.2 Does the child in the photo have any disease?	Does the boy in the photo look completely fine or is there something wrong with his body, head, mouth, somewhere?	**3.2.2** Do the ocular discharges have any relationship with a disease that you know?
	**3.3** Are there diseases that can spread from one person to another?	**3.3.1** Why can there be several people with the same disease at almost the same time? For example, when there is a flu or diarrhea... or what is seen in the child in the photo	
	3.4 The disease that the child in the photo has can spread to others. What should people do so that they do not get this disease?	**3.4.1** How can diseases be prevented from spreading from one person to another?	3.4.2 Is it enough to get into the pipe or the river to prevent one from getting or catching this disease?

**4. Identify the social networks on which their cultural identity is based**	**4.1** In families, who is the person who takes care of all the members of the household to know what to do when someone gets sick?	**4.1.1** If someone in the family becomes seriously ill, who is the person who knows When is it necessary to consult the PayÉ? Mom or dad, or someone else: grandma, grandpa...?	
	**4.2** To come to Mitú to have your eyes checked, did you ask someone for permission?		
	**4.3** To teach people in the community what western medicine does for the prevention of bad vision, what is the best time?	**4.3.1** Should we take advantage of Community Assemblies or Congresses or other community meetings?	**4.3.2** In which community meeting spaces should "Western" health actions NOT be carried out?
	**4.4** What is the good thing about what the trachoma people who have gone to the communities have done?	**4.4.1** What is wrong with the things that people with trachoma who have gone before to the communities do, talk about it and give them medication?	
**5. Identify motivators and barriers to participation in a System of Community Surveillance of the TT.**<br><br><u>intro</u>: All of you know from your own experience or from a close relative how much the evil eye that we call Trachoma hurts and you know of people who have even gone blind for this reason, and a blind person lives very badly, but those of us who are here today We could make this change. ... How?	**5.1** *Exercise:* Choose six people and place them in pairs, who should not know each other. Cover the eyes of one of them, ideally the oldest, and ask them to take a tour of the room with obstacles alluding to those of the jungle, first by the blindfolded person alone and then with their partner. How did the person who was left without seeing anything feel?	**5.1.1** What if he was really blind and found himself alone in the jungle?	**5.1.2** If you are blind, how would you try to improve your living conditions? Would you ask someone (family member or someone from the community) for help?
	**5.2** How did those who helped a person they didn't know feel so that nothing would happen to them?	**5.2.1** What good or bad things does helping other people that one does not know bring?	**5.2.2** There is someone who will never need the help of others people, your family, your friends and even others in your community?
	**5.3** How would you help a person who has evil eyes?	**5.3.1** Is it important to notify "Western" health personnel that a person has this disease? Because?	

SOCIO-CULTURAL ADAPTATION OF THE NATIONAL NEGLECTED TROPICAL DISEASES PROGRAM

Elimination of blindness due to trachoma in the indigenous communities of Vaupés

Focus group application for the intercultural construction of the information strategy, Education and

Communication-IEC, within the framework of component F of SAFE

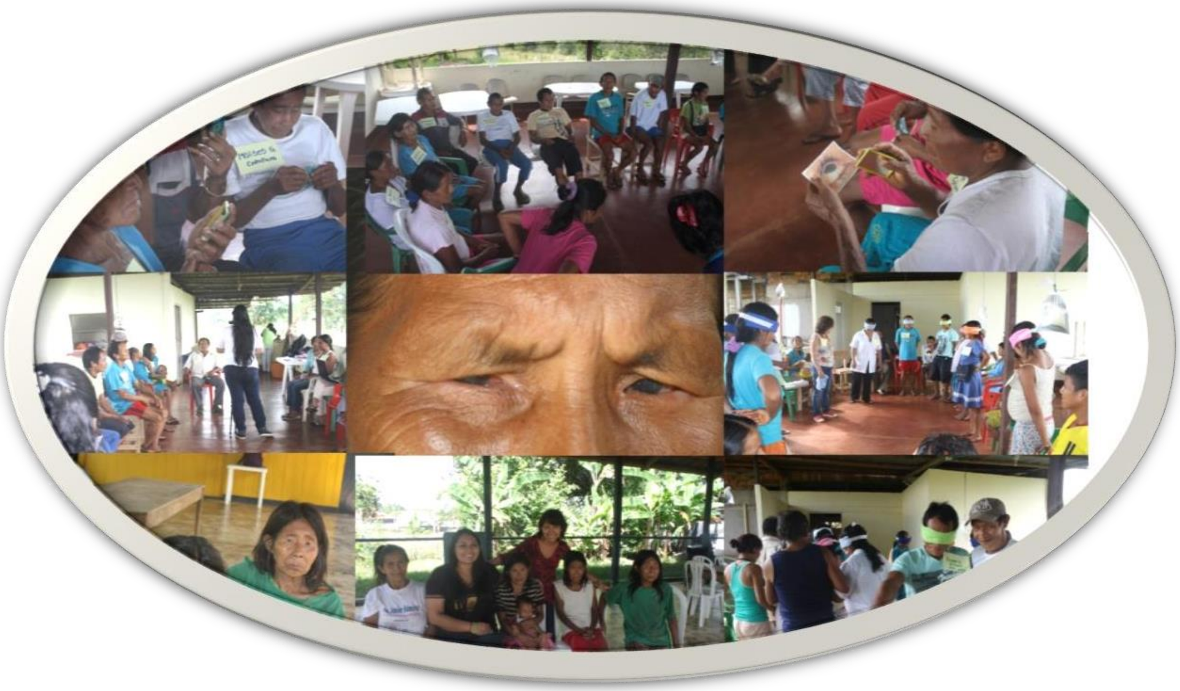

MINISTRY OF HEALTH AND SOCIAL PROTECTION

ALEJANDRO GAVIRIA URIBE

Minister of Health and Social Protection

FERNANDO RUIZ GOMEZ

Vice Minister of Health and Social Protection

ELKIN DE JESUS OSORIO SALDARRIAGA

Director of Promotion and Prevention

JOSE FERNANDO VALDERRAMA VERGARA

Deputy Director of Communicable Diseases

VAUPÉS HEALTH SECRETARY

INGRID TATIANA CORTEZ CAMARGO

Departmental Health Secretary

Report prepared by:

SOL BEATRIZ SANCHEZ MONTOYA

Contractor Management Promotion and Prevention

Ministry of Health and Social Protection

NOBODY NOVOA CHECK MARK

Social Communicator Secretary of Health of Vaupés

YURI CONSUELO RODRÍGUEZ RODRÍGUEZ

Reference Priority Chronic Noncommunicable Diseases Secretary of Health of Vaupés

MOISES BELTRAN MURILLO

Referent Community Participation Secretary of Health of Vaupés

PRESENTATION

The development of the focus group technique and the report of the results presented in this document are part of the study of knowledge, attitudes and practices-KAP, related to trachoma, in indigenous communities with a high risk of presenting blindness due to this cause. in the department of Vaupes. The application of this complementary qualitative information collection technique to this study is carried out with the purpose of obtaining information that contributes to the formulation of an Information, Education for Health - IEC and Social Mobilization strategy appropriate to the sociocultural context of the indigenous communities of this department.

INTRODUCTION

Trachoma is the first infectious cause of blindness in the world, the World Health Organization has set itself the goal of eliminating it, in the same way that the elimination of blindness due to Onchocerciasis has been achieved. Colombia adopted the goal (zero new cases of blindness due to trachoma in the year 2020), with the Neglected Infectious Diseases Program - EID, and with the implementation of the Comprehensive and Interrogant National Plan for the prevention, control and elimination of Neglected Infectious Diseases (2013-2017), in line with the Ten-Year Public Health Plan 2012-2021, the achievement of this goal is expected.

As part of the advancement of the goal for the country, the program has proposed formulating a communication- education strategy, agreed with the indigenous groups of Vaupés, given that the cases identified in the country at the moment are located in this population. As a preliminary exercise to the agreement of the strategy, the focus group technique was developed, within the framework of the Third surgical day for the correction of trachomatous trichiasis and the first of the social mobilization of patients, in the city of Mitú, capital of the department. This document containing the results of this qualitative exercise will then be included and related to the results obtained from the application of the CAP surveys.

The information related here is developed in parts. Part I integrates the justification, characteristic, objectives, the purpose of the focus group. In Part II, a cultural approximation of the ethnic groups that participated in this exercise is made, with some population data. In part III, the methodology of how the focus group was developed is described. In part IV, the systematization of the information and its analysis are presented. And part V is the closure of the document with some conclusions and recommendations. It should be noted that all the primary information collected in videos, audio recordings and photographs, etc. is attached to this report.

PART I

CONCEPTUAL JUSTIFICATION OF THE FOCUS GROUP

The focus group is a technique for collecting and constructing qualitative information collectively, in the same way as other strategies, techniques and instruments used in qualitative research. The focus group aims to approach the subjective experience of the participants, both individuals and groups, that is, the feeling, thinking and expression of the subjects within a specific cultural context:

“The focus group has epistemological foundations in Qualitative Social Research, with a 'constructive- interpretative knowledge' character: obtaining data through focus groups is characterized by the value of the singular, as 'instances of production of knowledge'. scientific knowledge' for which social significance is achieved.”

From a constructionist perspective, reality is a social construction that occurs through interaction and social practices. The language of the actors allows this construction while bringing the researcher closer to understanding it.

Therefore, it is essential to approach the recognition that knowledge or "popular knowledge" is so valuable as, the so-called scientific knowledge, since it implies the knowledge of a social reality from the subjects themselves and contexts where their daily life is expressed.

Hence, the focus group becomes a very useful tool to collect both knowledge and perceptions, sensations, practices, among other aspects, that people live on a daily basis in the face of a particular problem, aspects that are key inputs for the planning of programs and their evaluation.

CHARACTERISTICS OF THE FOCUS GROUPS

Unlike the group interview, the focus group focuses on the interactions that occur within it around the topic that the researcher has proposed, hence the social dynamics becomes relevant as a way of producing collective knowledge. On the other hand, the group interview focuses on the interaction of questions and answers, by the interviewer and the interviewees, while, in the focus group, the participants express themselves freely and openly on certain topics of interest, and exchange their ideas. around them.

However, to carry out this technique, participants with homogeneous characteristics and common experiences must be selected; between 6 and 12 people; establish clear objectives, as well as their scope, and from there prepare a list of questions or key issues to be addressed; the moderator must have the ability to promote interaction between the participants, be open, respectful of the word, flexible, and especially, although he guides the participation, he must intervene little in the dynamics; have technological tools to record information, as well as the support of other people to record field notes.

PURPOSE APPLICATION OF THE FOCUS GROUP DISCUSSION TECHNIQUE FOR TRACHOMA

The purpose of the application of the focus group technique as a qualitative research instrument was todeepen the knowledge, attitudes and practices that the indigenous communities of the department of Vaupés have about trachoma, as well as the conceptions that these Communities deal with hygiene, cleanliness (personal and collective), health, illness, how they prevent illnesses, how they cure illnesses, their concept of care, protection of the individual, the collective, the home and the territory, their individual and collective forms of relationship (intra-community and outside the territory), and their forms of communication. This as a necessary input to understand the dynamics of these communities and thus achieve the collective construction of an IEC strategy (Information, education and communication).

GENERAL OBJECTIVE

The general objective was oriented towards the collection of inputs for the design of an Information, Education and Communication (IEC) strategy, aimed at the prevention of trachoma, and a plan for community surveillance of trachomatous trichiasis and blindness due to trachoma with participation of patients, appropriate to the cultural and social context of the indigenous population of the department of Vaupés.

SPECIFIC OBJECTIVES

The specific objectives were defined as follows:

1. Identify behavioral change objectives related to trachoma transmission in focus groupparticipants, for the design of the IEC strategy.
2. Identify the concepts of hygiene, cleanliness and their relationship with the disease, among the study participants inrelation to their worldview.
3. Identify the social networks on which their cultural identity is based, to establish the bases on which the IEC strategy and the Community Surveillance Plan for trachomatous trichiasis and blindness due to trachomashould be built.
4. Identify motivating elements and barriers among the participants to design and implement a community surveillance strategy for trachomatous trichiasis and trachoma blindness in the department of Vaupés.

PART II

CULTURAL APPROACHES OF THE INDIGENOUS REPRESENTATIVES PARTICIPATING IN THEFOCUS GROUP

The total indigenous population of the department of Vaupés is 15,8898.2, that is, 70.44% of the population of the department(30,529) and 1.43% of the indigenous population of the country (1,106,499). In its territory there are five indigenous reservations: Bacatí-Arara, Yaigojé-Río Apaporis and the Eastern Part of Vaupés, the latter divided into three parts corresponding to the jurisdiction of the Mitú and Carurú municipalities and the Yavaraté departmental corregimiento, with a total area of 4,120,897 hectares, that is, 63.14% of the area of the department (6,526,800 hectares) and 13.36% of the total indigenous area (30,845,231 hectares). The reservation with the largest extension is the so-called Eastern District of Vaupés (3,354,097 hectares), which occupies 81.39% of the total area of the three reservations located in that department (4,120,897).

Below is a brief cultural approximation of the 14 indigenous peoples that had representatives present inthe focus group, based mainly on the reviews prepared by the Ministry of the Interior from secondary information sources:

1. THE TUCANOS

> “Within his world view, the universe is understood as a static crystal ball made up of stars, planets, hot and cold. Ñeku, ancestor of the Tucanos, discovered the powers of the universe,food and the beings that inhabited it.

They call themselves "Dahséamahsá", the toucan people. They live on the border between Colombia and Brazil, in the townsof Acaricuara, Montfort, Piracuara and the hamlets of the Paca, Papurí, Tiquié and Guaviare rivers. The life of this town sincethe conquest, has been impacted by the imbalance given from the inter-ethnic contacts with settlers and missionaries. The extractive industries that were generated in the Amazon region, such as the exploitation of rubber, led the Tucanos to resist situations of slavery, displacement and epidemics for centuries.

Among the main characteristics of this culture is the practice of exogamy, since marrying someone who speaks the same language is considered incest. The Tukana language, potentially threatened, is acquired through the paternal-filial line.*“There are several magical-religious specialists among which the Ku'mú – thinker stands out”.* However, the highest political authorityis the 'Captain'.

In relation to your home, the following is highlighted:

> "Although they currently live in single-family houses grouped in villages, in some communities there are malocas that function as political and cultural meeting places. The symbolic association between chagra, kitchen and house, articulates his notion of territory. The kitchen is made in a construction attached to the house and is identified with a bower and a wood stove.

The basis of their traditional economy is based on the practice of itinerant horticulture, from the slashing, slashing and burning of trees. This activity is complemented by fishing, hunting and gathering, as well as the cultivation of fruit trees in their fields.

Table 1. POPULATION DATA OF THE TUCANO PEOPLE			
	**Man**	**Women**	**Total**
DANE data, according to the 2005 census	983	1033	2016
Data according to Life Plans of the Tucano People	1117	996	2113
Data according to the census list trachoma survey?			

2. THE BARÁ

> "In recent years, insufficient studies have been carried out on the trajectory of this group or on its current situation. However, they have been classified in ethnography as part of the so-called Vaupés cultural complex, a characteristic that makes them similar to other groups as close, belonging to the Tucano Oriental linguistic family such as the Tatuyo, Desano and Wanano".

The fish people. They live along the Colorado, Yapú, Papurí, Inambú, Macucú, Tiquié and Vaupés rivers on the border with Brazil. By the 1980s, the Bará lived in malokas and nuclear villages of 12 to 60 people. Possibly at present, common forms of settlement have been adopted among other towns in the region, such as the grouping of houses around the maloka, the school and the soccer field.

They follow an exogamous and patrilineal pattern, with prevalence of monogamy. In relation to their worldview, it is said thateach animal species has its own maloka and an owner. Once the person dies, the soul travels to the maloka of his ancestors: *"The maloka is for the exclusive use of the people, for this reason those who do not consider themselves fully human, such as newborns or those bitten by snakes, cannot enter until the Shaman, a figure of great importance in the community, enters. do not grant them this condition"*

**Table 2. POPULATION DATA OF THE BARA PEOPLE**			
DANE data, according to the 2005 census	**Man**	**Women**	**Tota l**
	52	48	100
Data according to Life Plans of the Bará People	168	153	321
Data according to the census list trachoma survey?			

3. THE PIRATAPUYOS

Children of fish and/or gű̃io o boa They live in the Bajo Papurí region, some live near Teresita (currently thereare some families living in the towns of Meta and Bogotá). About its origin the following is related:

> "The origin of the Piratapuyo (Wai'kana) begins in the milk lake, that is, Bocas del Río Negro (sea). Formerly there was only one GOD DEPARICOACU (LAZY GOD) looking that there were no people he wanted to create, he took out two totumos, in those contained yópoca (corn granules) in another ujetá po'cá (starch granules) because at that time there was no *mambiada* or I pray. After 5 days he threw it into the lake, many fish came out to eat and they finished everything. At that moment inside the water there was noise, people's screams, looking at that he prayed again and threw into the lake, that day they also came out they ate everything and people were born at night and during the day they returned to the lake; Thethird time he prayed: first he organized a maloca with all the musical instruments, chicha… they left at 5:00 pm and came upfrom the river. Each one had their benches to sit in the maloca. They drank chicha until dawn. It is the reason for the name Wai'kana, because they came out of the fish. After all that, they embarked with PAMORIYUCUSA (guio canoe) up the

Negro River, they went up all the sacred places, crossing the Vaupés river until they reached Santacruz, where the Cubeos were already. That's why they went down again to the line from Tucunaré arriving at the Osomucjuriña stream, from there they went up to Oaripawu and stayed there for a while. Then they went down to Canaña (Caño Macucu) this was looking for the exact place where they were going to stay, they measured with the baton and it did not mark well, they went down to Wainambí (Imi dapu) there were already people, from there they went down to Meyú (San Antonio) again they went down to San Francisco (Tuoturuco) they stayed for a while, then they continued their journey to the mouth of the Wasay, from there they went up until they reached the head of the Yacaré channel up to Suspiro hill, there they stayed[7].

“They call themselves Dokapuara, the 'clay people.' Within their cosmology, the Tuyuca descend from the Anaconda Piedra or Cachivera. They consider the Tucano and Bará as their traditional allies.”[8]

**Table 3. POPULATION DATA OF THE PIRATAPUYO TOWN**			
DANE data, according to the 2005 census	**Man**	**Women**	**Tota l**
	There is no data	There is no data	0
Data according to Life Plans of the Piratapuyo People	433	339	772
Data according to the census list trachoma survey?			

4. THE TUYUCAS

Clay people. They live along the Inambú and Tiquié rivers. In Papurí, south of Acaricuara, there is a community. They belong to the Eastern Tucano linguistic family. They have a patrilineal filiation pattern, they are exogamous and monogamous.

The Captain is the main authority of the community, he is the one who represents it before the institutions and other communities, and in the exercise of his functions he is accompanied by other leaders. On the other hand, there is the Payé, who fulfills the function of protecting and guiding the Tuyucas, and is the one who holds the power to face supernatural forces. In addition, the Payé exercises traditional medicine together with the Cumú:

> "The payé is the traditional doctor of the community who acts on the members of their communities from the birth of children, growth stage, initiation rite of young people, in diseases, dangers, dances, festivals and in other special moments. He prays or blows the parents' food when a child is born. He prays to the children and places a cultural name on it. Pray for yagé, a traditional drink in dances, coca and cigarettes for medicinal purposes, as well as 'el breo', scented wax that some bees make and that when placed on a pot with burning embers the smoke is spread for charitable purposes for the community. He also prays food after dances, to the sick, and on other occasions. The Cumú is who prays, who prays, and is called to perpetuate moral teachings. Its function is to maintain union and stability, prevent disease and also intercede with the supreme forces, the owners of nature, heaven, earth, animals, for someone's good. The Bayá or dancing singer is the one who translates the relationships with the supreme into dances and songs and directs the dances with the community at parties.

The base of their diet and their main crop is cassava brava. They practice slash-and-burn horticulture, along with fishing, hunting, and gathering wild fruits. Likewise, they make crafts expressed in woodwork, basketry and pottery. They barter their products for other necessary household items.

**Table 4. POPULATION DATA OF THE TUYUCA PEOPLE**			
DANE data, according to the 2005 census	**Man**	**Women**	**Total**
	229	215	444
Data according to Life Plans of the Tuyuca People	438	414	852
Data according to the census list trachoma survey?			

5. THE DESANOS

"Although not enough studies have been done on the current situation of the group, ethnography describes the 'Yuruparí' ritual as the primordial axis around which they remember their origins and reaffirm the links with their worldview."

Lightning people. They are in the Abiyú channel, the Macú Paraná channel and in Piracuara (the Virarí channel). There are some who live by the Vaupés river. They practice a self-subsistence economy, based on fledgling horticulture, hunting, fishing, and gathering. Among its main crops are bitter yucca, sweet yucca, sugar cane, corn, chontaduro, yams and some fruit trees. They make reed baskets and clay pots, standing out as skilled craftsmen.

Although they previously lived in malokas, it seems that they currently follow the settlement pattern common to other peoples in the region, locating their homes around the maloka. Their social organization responds to a hierarchical system, divided into patrilineal lineages. Among its main authorities are the Payé and the Cumú: *"The Payé officiates the life cycle ceremonies, cures illnesses, mediates between supernatural forces and deals with the 'owners' of hunting animals. The Cumú who is considered a representative of the sun, is the person with the highest rank within society.*

**Table 5. POPULATION DATA OF THE TOWN OF DESANO**			
DANE data, according to the 2005 census	**Man**	**Women**	**Total**
	1093	1083	2176
Data according to Life Plans of the Desano People	1139	1046	2185
Data according to the census list trachoma survey?			

6. THE SIRIANOS

Cloud people. They are along the Paca channel, a tributary of the Papurí and along the Viña river, a tributary of the Paca; One group lives at the mouth of the Ti channel, which empties into the Vaupés.

**Table 6. POPULATION DATA OF THE SIRIAN PEOPLE**			
DANE data, according to the 2005 census	Man	Women	Total
	There is no	There is no	0
Data according to Life Plans of the Sirian People	532	514	1046
Data according to the census list trachoma survey?			

7. THE TATUYOS

Armadillo people. They are located in the upper part of the Pirá Paraná river and its tributaries. They are considered one of the most traditional towns in the Amazon region,

since they have apparently remained relatively isolated from urban sectors: *“The current pattern of settlement in the Papurí area includes small villages, some malokas, and a large missionary town in Araracuara.”*

As with other indigenous peoples from this region, the Tatuyo have a patrilineal filiation and residence pattern. Its main political authority is the 'Captain'. Socially they are organized in clansdefined from tradition:

“The fundamental segmental units have been clearly identified as clans or sibs; of little genealogical depth, the clan is made up of one or more patrilineages that are recognized as common descendants of a mythical ancestor without being able to trace the genealogical ties back to its founder; the nomination is eponymous, but in certain cases the reference can be established by the place of origin on the river where their ancestors arose.

Being hierarchically organized according to the birth order of their founders, they are considered classifying brothers and therefore, an exogamic unit; the order of distribution of their local groups on the river reproduces the social hierarchy, living in areas close to the place of emergence of their ancestors. Personal names are passed down according to each clan. A clan or a group of such is associated with the exercise of specific functions that discriminate tasks such as captains, singers, dancers, shamans, warriors and servants; such tasks comprise activities that are distributed among clan members. The ordering from highest to lowest guaranteed the ascription of the rank and thespecific function.”

They practice the self-subsistence economy, through horticulture, hunting, fishing and the domestication of animals. Likewise, the cultivation of cassava, taro, pineapple, banana and chili, is characteristic of their fields. They are skilled inhandling fibers for making basketry.

Table 7. POPULATION DATA OF THE TATUYO PEOPLE			
	Man	Women	Total
DANE data, according to the 2005 census	189	192	381
Data according to Life Plans of the Tatuyo People	317	270	587
Data according to the census list trachoma survey?			

8. THE CARAPANS

“Like other groups such as the Bará, Barasana and Desano, the Carapana are part of the Vaupés cultural complex, a classification that has been given to them in ethnography based on the similarities in their way ofexploiting the territory, in their systems of social organization and its mythical descent, among other aspectsof its worldview.”

Walking people. They are scattered along the Tí, Pirá Paraná, community of San Antonio (Papurí) and Vaupés rivers. Formerly they formed a single family with the Taiwanos, considered as the younger brothers. They live in houses made from different kinds of palm trees occupied by nuclear families.

The Carapana practice slash-and-burn horticulture, as well as hunting, fishing, and gathering fruits and insects. Its main product is cassava brava. Traditionally, the main authority was the "Jefe" supported by the Payé.

**Table 8. POPULATION DATA OF THE CARAPANA PEOPLE**			
DANE data, according to the 2005 census	**Man**	**Women**	**Total**
	220	218	438
Data according to Life Plans of the Carapana People	372	300	672
Data according to the census list trachoma survey?			

9. THE MACUNAS

"According to their myth of origin, the world and the 'Yuruparí' were created by Romi Kumu, the Shaman woman, bodily identified with the earth. For its part, the Anaconda de Yuca considers itself the owner of animals, wild fruits and all other elements that inhabit the world."

People water. They live in the community of Piedra Ñi, located on the Pirá Paraná, as well as on the banks of the Comeña River, a tributary of the former, and on the lower Apaporis, south of the Vaupés. The Macuna practice slash-and-burn shifting agriculture. The main product they grow and consume is cassava brava, which is complemented by the cultivation of a variety of tubers, fruit trees, and plants used in rituals. The women manage the chagras, except for the cultivation of tobacco and coca, which corresponds to the men. In addition, they practice hunting, fishing and gathering. Among the main spiritual figures, the Kumu-thinker stands out, followed by the Yai (traditional doctor). Socially they are organized into seven groups related to each other, grouped in turn into two exogamous fatrias (they are made up of clans organized according to an order of ancestry): "Ide Masa and Yiba Masa. The exchange of women is made between both fratries. The most numerous groups are the Ide Masa, known as -The People of the Water-, which has the highest rank within the total structure of the group."

MINSALUD

Table 9. POPULATION DATA OF THE MAKUNA PEOPLE			
DANE data, according to the 2005 census	**Man**	**Women**	**Total**
	157	149	306
Data according to Life Plans of the Makuna People	515	408	923
Data according to the census list trachoma survey?			

10. THE BARASANS

> "The old people say that in the beginning of humanity the anaconda snake went up the river and left the different groups that today live in the Vaupés jungle. Since then, the Barasana of Pirá-Paraná have lived in the jungle, gradually discovering its secrets, without destroying the life of plant and animal species... They are people of canoes, harpoons, traps and hooks. Among the trees of the jungle they learn to choose the one that they will transform into a canoe; They are fishermen, they know which are the favorite places of the fish, what bait attracts them and when they come close; They are also hunters, using bows and arrows, blowguns, and shotguns. They know the life of the animals in the forest."

Fish people. They live in the central area of the Pirá Paraná and its tributaries. In addition to hunting, fishing, and gathering insects and wild fruits, the Barasana practice slash-and-burn horticulture. Its main crop is cassava brava, and this, as well as its derivatives, are a fundamental part of the daily diet. Other crops are corn, sugar cane, pumpkin and plantain.

The men's work consists of preparing the land, fishing and hunting, as well as making handicrafts; For their part, the women are in charge of keeping the chagra clean, harvesting and preparing food. Regarding its social and political organization, the following stands out:

> "Their social organization is characterized by a strong kinship network; they exchange women with the Bará and other nearby communities. Traditionally the main authority is the head of the maloka, however, there are other characters who fulfill religious functions such as the Payé, the Cumú, the specialist in songs and dances and the myth recitation teacher. They consider themselves allies of the Makuna."

Table10. POPULATION DATA OF THE BARASANO PEOPLE			
DANE data, according to the 2005 census	**Man**	**Women**	**Total**
	twenty-one	29	fifty
Data according to Life Plans of the Barasano People	462	429	891
Data according to the census list trachoma survey?			

1. *Idem.*
2. http://siidecolombia.gov.co/sites/default/files/upload/SIIC/PueblosIndigenas/pueblo_barasano.pdf.Pág. 3
3. *ibid.*Page 4

11. THE CUBEOS

"Within its worldview, its origin is associated with the mythical cycle of the Ancestral Anaconda. Kuwai is the main cultural hero and the 'Yuruparí' ritual stands out among its celebrations."

Children of Kubay. They live on banks of the Vaupés River from Santa Cruz de Waracapuri upwards and dominate the large tributaries of the Vaupés, such as the Querarí and Cuduyarí rivers and the Cubiyú and Carurú streams. Like other towns in the region, they practice slash-and-burn horticulture, growing cassava, fruit trees, and other tubers. Likewise, they practice fishing and within their diet the consumption of insects and wild fruits stands out. Some of its products are destined for commercial exchange, such as fariña, which is a product of yuca brava, as well as corn, meat and fish.

The characteristic of their settlement pattern is the conformation of dispersed villages around a school or a health center. Likewise, in some villages there are malocas whose function is to house visitors, and of course, they are places of meeting and ritual practice.

Regarding the social and political organization of the Cubeo people, the following is affirmed:

> "The social structure of the Kubeo group is characterized by the Sibs, groups that do not have a defined leader. Each of these groups has a particular origin in the mythological world, as well as particular ancestors or 'grandparents'. These Sibs are exogamous, patrilineal and patrilocal units. Between the Kubeo and Makuna there is a marriage exchange with geographically close groups. The basic nucleus of each village is made up of a group of siblings related through the father's way... Politically they have two types of chiefs: the haboku or man of goods and the kenámi up.ku or owner of the house."

The main traditional authorities are the Payé and the Curandero, who manage the rituals aimed at maintaining the balance of the spiritual world, its protection and restoration, using chicha and yagé.

**Table 11. POPULATION DATA OF THE KUBEO PEOPLE**			
DANE data, according to the 2005 census	Man	Women	Total
	1750	2006	3756
Data according to Life Plans of the Kubeo People	3243	2991	6234
Data according to the census list trachoma survey?			

12. THE JUPDAS

They live in the area between the Papurí and Tiquié rivers. They practice hunting using blowguns, darts with curare and bow and arrow, and gathering insects and animals such as frogs, as well as honey, fruits and tubers. Likewise, they practice itinerant horticulture on a small scale, where products such as cassava, yams, fruit trees and coca predominate. Originally, they were nomads, for which reason they were designated by other peoples with the name "macú".

They move mainly on foot through the region, unlike other peoples who travel in canoes. Their homes are characterized by constructions without walls, with a thatched roof made of palm leaves. Traditionally they live ingroups of 20 to 35 people, which is equivalent to an average of six families.

The Shaman or Payé, is the spiritual authority of the Judpá, in charge of the protection of health. One of the most important celebrations of this town is the Dabukurí, where they celebrate reciprocity through the exchange of giftssuch as wild fruits, meat, fish, fariña; In the event there is dancing, singing, playing flutes and drums, and consumption of chicha or fermented cassava liquor.

Regarding the social organization of the Chupdá, the following refers:

> “A set of local groups at a distance of one day's walk from each other, form a large regional group. The Jupdá make up at least three regional groups, of about 260 people each, separated from each other by navigable watercourses, whose banks are occupied by "river Indians" (mainly Tucanos). Adults from the same regional group all know each other by name and know precisely the relationships of kinship that unites them, which contrasts with the precarious knowledge they have of the people of groupsfrom other regions... The regional group is endogamous, while within it, each clan is exogamous since marriage or relationships are considered inappropriate sexual relations between people of the same clan. This correlates with a Dravidian-type kinship terminology that regards cross-cousin marriage as ideal…and instead prohibits parallel-cousin marriage.”

Due to the progressive colonization of their territory, causing negative impacts on the environment and, therefore, health problems, the Jupdá have tended to become sedentary, causing new problems regarding their living conditions.

No population data has been found, neither according to DANE, nor in the life plan.

13. THE ITANOS

They live around the Cachivera de Coro, Pirá Paraná river.

14. THE YUJUB

They are societies in the process of sedentarization because a short time ago they were hunters and gatherers. They predominate inthe Tiquié and Bajo Apaporis areas. In a derogatory way they have been called Macú.

PART III

DESCRIPTION OF THE DEVELOPMENT OF THE FOCUS GROUP

The data of the qualitative component (focus group) were collected based on a guide developed for this purpose. Initially, it was expected to form two groups, one for men and the other for women. But in the development, it was only possible to form a mixed group of 21 people (13 women and 8 adult men) as stated in the list of participants that is attached to this report.

The people who were included in the focus group were guaranteed transportation to the city of Mitú from their communities of origin, with their relatives or companions, as well as shelter and food, and at the end they were given a present that consisted of a hammock and kitchen utensils, as an incentive for their participation and collaboration.

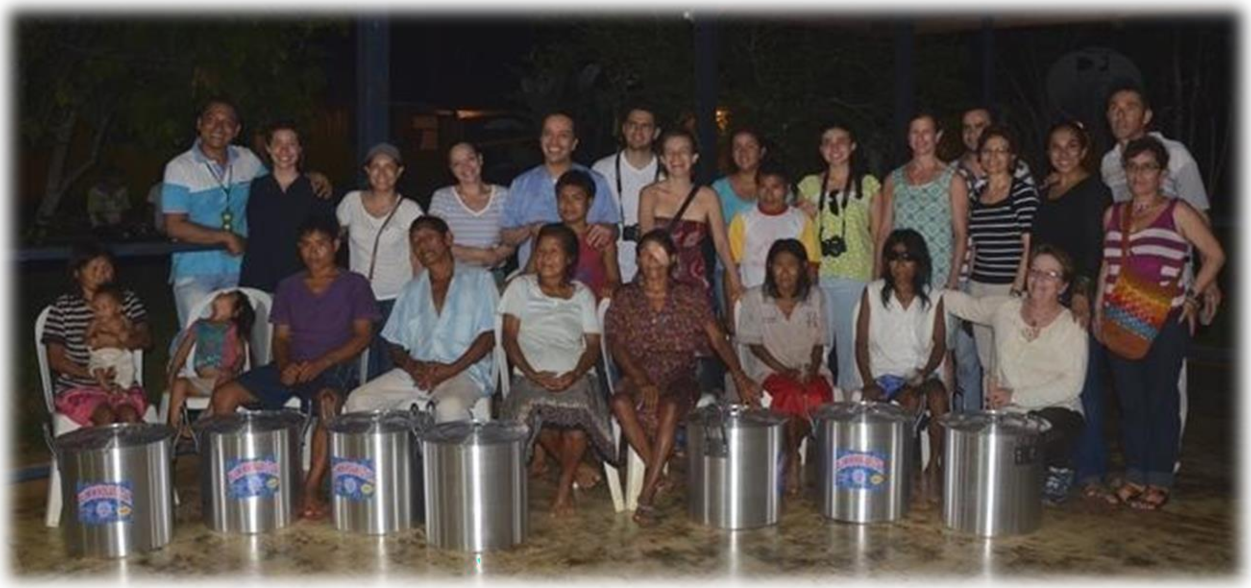

Photo No. 1 Delivery of presents to trachoma patients, Catalina Cárdenas, social communicator-PAHO, May 2014.

A group of consultants from the National EID Program (Neglected Infectious Diseases) of the Ministry of Health and Social Protection, and from the MSPS/PAHO Agreement were in charge of coordinating this exercise and applying the guide for the development of the focus group and analyzing the information collected through this qualitative exercise.

Considering that the focus group was made up of representatives of various indigenous peoples who spoke different languages, there were bilingual translators, mainly for Tucano and Cubeo, which are the most dominant languages in the department. The translators were: Rebeca Mendoza of Cuban origin, María Imelda Arango Andrade, Rogelio Peña of Tucano origin and Cecilia Jaramillo of Barazana origin.

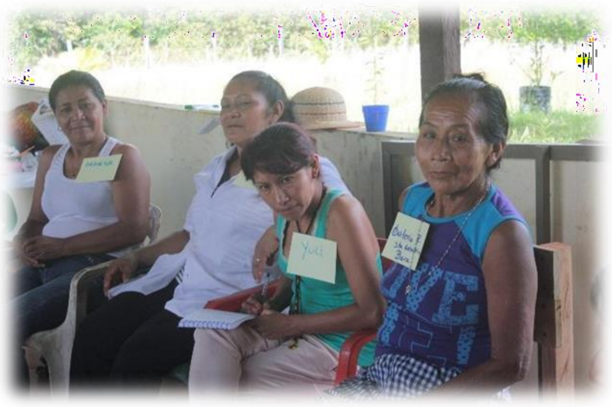

Photo No. 2 Translators focus group
Photo: Nadezhda Novoa.

The information was collected through discussion-generating questions and suggestive images of situations related to the explored aspects.

The development of the work of the focus group was supported by two officials from the Secretary of Health of the Department of Vaupés and an official from the Ministry of Health and Social Protection, to fill the following roles:

1. Moderator: application of the guide to direct the discussions around the topics of interest and prevent them from straying from the fulfillment of the objectives of the study.

2. An observer and rapporteur: This person is an official of the Territorial Directorate of Health of Vaupés. The function of this participant was: Record the relevant aspects of the global behavior of the group, seeking to identify group or individual situations and attitudes that provide non-verbal information related to the knowledge, attitudes and practices that are being explored.

3. Recorder: Responsible for carrying out the video and photographic recording of the entire work session. The photographic and audiovisual record is attached to this report.

Materials used for the development of the focus group:

The materials used were chosen based on the dynamics developed during the application of thistechnique as listed below:

- Photographic camera
- Video recorder.
- Tape recorder
- Photo healthy eyes and eyes with trachoma and child with diseased eyes
- Mirrors
- News paper
- Cardboard.
- Colors
- Markers
- hand towels
- masking tape
- Meeting space in the House of passage EPS CAPRECOM agreement.

FOCUS GROUP WORK DYNAMICS

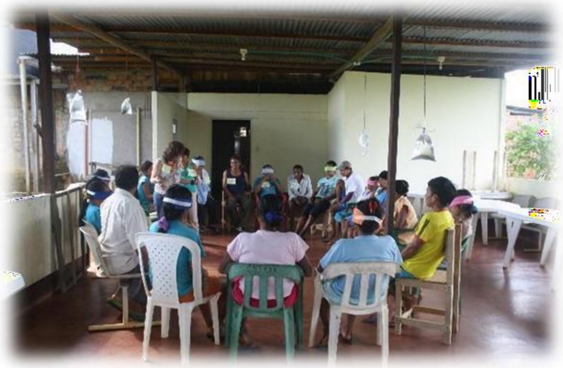

Photo No. 3 Lazarillo and the blind man dynamics

As a data collection instrument, a guide of motivating questions was prepared, which was included in thedynamics described below:

To inquire about the knowledge that the participants had about trachoma, as an introductory exercise, the mirror dynamic was used, which consisted of giving each one a small mirror so that they could observe their eye and then describe what theysaw. Next, each one was given two photographs, one of a healthy eye and the other of an eye suffering from trachoma, and their perception of the situation of these two eyes was addressed. It should be noted that for many the photo of the healthy eye was also considered as a diseased eye, due to the high vascularity present in the photographed eye. Therefore, another strategy had to be used to achieve the compare and contrast exercise.

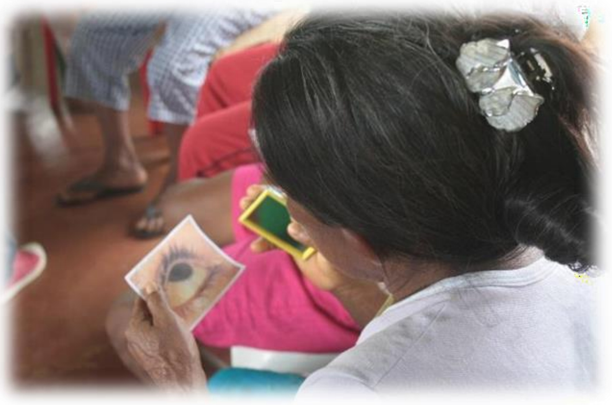

Photo No. 4 Exercise seeing the eye

In a second moment and with the aim of getting closer to the understanding of hygiene and cleanliness, an exercise ofobservation and description was worked on based on a photograph of a child with a face full of snot and rheumy eyes.

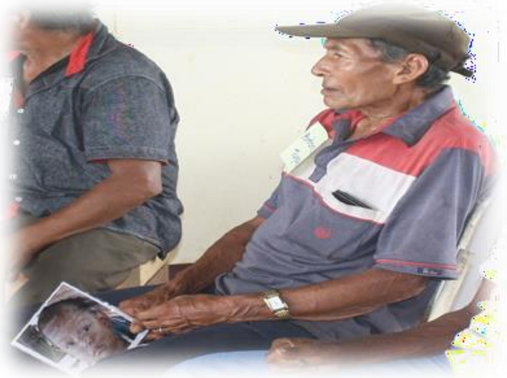

Photo No. 5 Exercise watching children with sick eyes

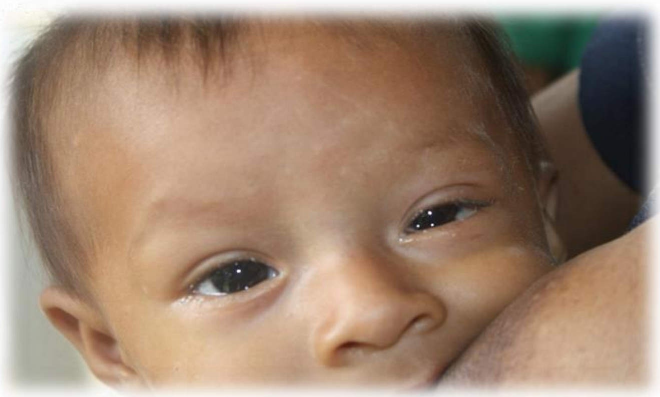

Photo No. 6 Child of the Yujup ethnic group with a problem of conjunctivitis

There was also a dramatized about the practice of a bad facial cleansing and a good facial cleansing.

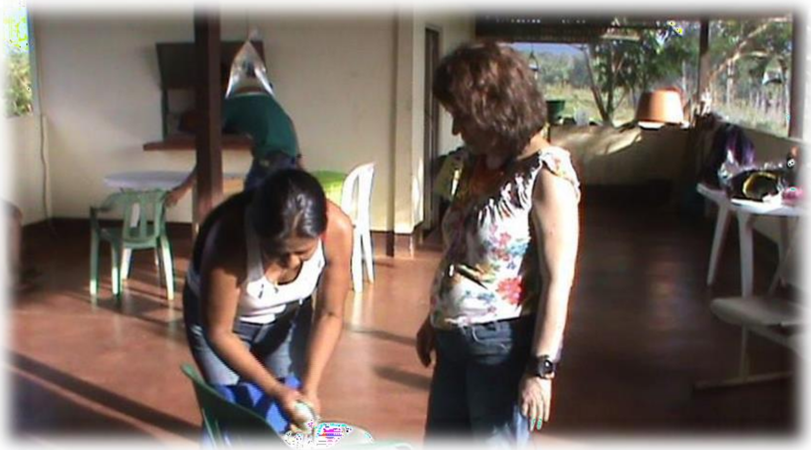

Photo No. 7 Facelift exercise

To introduce the discussion of the prevention of blindness due to trachoma, the dynamic of the guide and the blind was developed, which consisted of making groups in pairs, one blindfolded representing a blind man and the other representing the guide, the person with the eyes blindfolded. Blindfolded, he could shake hands with the guide or put it on his shoulder, it was done in a closed space, clear of objects. Then the roles were switched. At the end, each one of the participants expressed their perceptions about blindness and living with blind people in their communities.

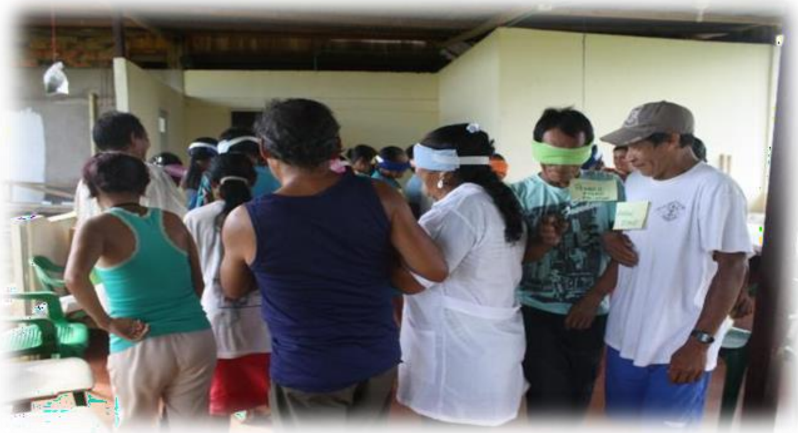

Photo No. 8 Dynamics of the guide and the blind man

Active breaks were made through integration dynamics, which allowed the interest and attention of the participants to be maintained until the end of the day.

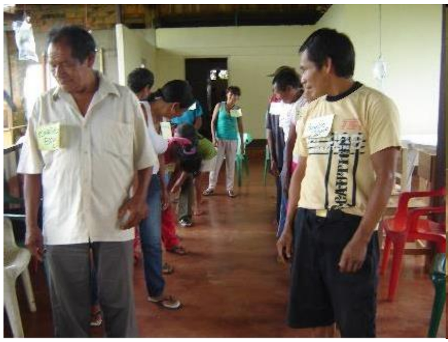

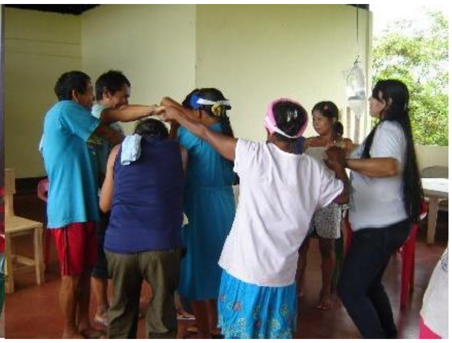

The previous dynamics gave the possibility of introducing questions related to the identification of the knowledge that the participants had about trachoma, prevention, treatment and origin.

What do you wash your face and hands with? How often? Why do you get sick eyes? and how are they cured? How are the eyes cared for and protected? How do they communicate within the community? How do they communicate with other communities outside the territory? How do they relate between families? What would a

good call be like? How do they care for their family, the community?), How do they help each other? Willingness to work with health institutions on trachoma?

PART IV

SYSTEMATIZATION AND ANALYSIS OF THE RESULTS OBTAINED FROM THE APPLICATION OF THE FOCUS GROUP

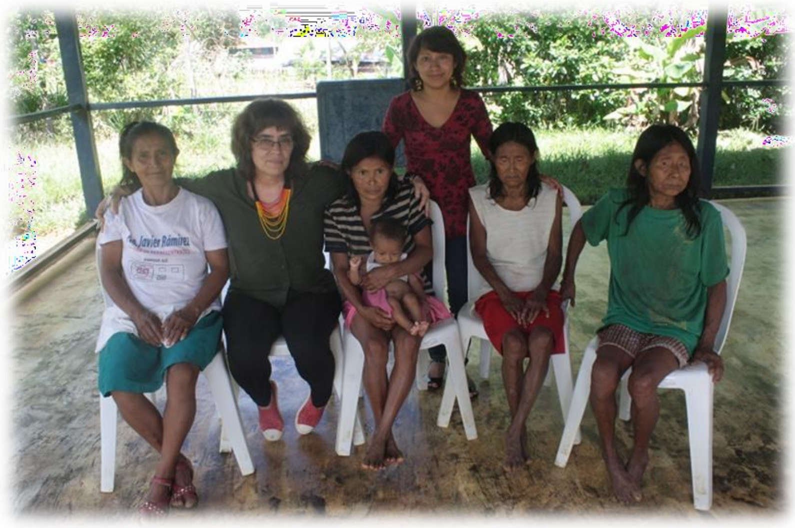

Photo No. 9 Women of the Yujub ethnic group

SYSTEMATIZATION OF THE INFORMATION

The exercise of the systematization of the information obtained during the development of the focus group, starts from the deductive and inductive concepts as categories of analysis, as explained below:

The different questions that are part of the tools used, are configured as deductive categories of analysis which respond to the information needs against the design of the strategy. These categories arise from deductive reasoning, which:

> "… it is useful for research, it offers resources to unite theory and observation, in addition to allowing researchers to deduce from theory the phenomena to be observed. The deductions made from the theory can provide hypotheses that are an essential part of scientific investigation.

The deductive categories become the starting point of approaching the reality that is intended to be known and understood, and of course, through the analysis they have been related to the categories that arise inductively from the field work through the development of the focus group

or inductive categories. The latter arise from inductive reasoning: "...*in deductive reasoning the premises must first be known before a conclusion can be reached, while in inductive reasoning the conclusion is reached by looking at examples and generalizing from them to the whole class*."

In summary, compared to deductive and inductive reasoning, from which the respective categories of analysis are derived, it can be estimated that:

> "Deductive and inductive reasoning is very useful for research. The deduction allows to establish a link between theory and observation and allows to deduce from the theory the phenomena object of observation. Induction leads to the accumulation of isolated knowledge and information."

The complementarity of the two reasonings that are translated into analysis categories is of vital importance for understanding the problem to which the application of the focus group technique seeks to respond. The exercise is presented below.

Information systematization matrices:

Matrix 1.

**CATEGORY deductive**	**TESTIMONIALS**	**INDUCTIVE CATEGORY**
Knowledge about trachoma<br><br>trachomatous trichiasis	(Use of the mirror) "To remove the eyelashes thatget into the eye, to see the eyes and remove the dirt that gets into the eye."<br><br>Rogelio: "the eyelashes are lowering towards the eyes, the state of the eyelids, they indicate that it iswrong"<br><br>"This disease is not normal; the trachoma disease is a curse that the same people create."<br><br>in some communities there is a lot of envy for what they have, that's why we hurt ourselves".<br><br>"This disease is incurable forus, when the disease starts in children it causes blindness, sometimes doctors cure the disease, but in other cases they don't.	Recognition of the disease (eyelid invested).<br><br>Curse caused by envy.<br><br>Incurable disease in some cases.<br><br>It can cause blindness.<br><br>norm transgression culture related to food (from the symbolic).
	"In addition, there are several cultures that do not take care of the culture, when they eat mojojoy fromthe pupuña palm or from those that have a thorn, they produce trachoma disease. the palm mojojoy that do not have thorns can be eaten."<br><br>Rogelio: "Loss of sight is evil, it's different fromtrachoma disease", it used to occur, now it doesn't, because the people who knew about this evil have already died. "It's an inexplicable mystery, other people think it's amazing, that's respectable."<br><br>"What we've heard is called oculartrachoma."<br><br>"All diseases are called eye pain, but when white dots occur, when vision is lost, it is called white dot eye. But that has its cure, we have tried with traditional plants", but this trachoma disease is different, it has no cure.<br><br>"Trachoma occurs at any time, so it cannot be called the age of healthy eyes."<br><br>"conjunctivitis and tooth pain according to the Payé appear at the time of the chontaduro and the time ofthe tapururus worms.	Inexplicable mystery.<br><br>Whiteheads on the eye:Whitehead eye that is treated with floors traditional vs. Disease of the trachoma (it has no cure).<br><br>It can be submitted at any time.<br><br>Presence of diseases According to epochs (calendar ecological).<br><br>Not symptoms.<br><br>Contagious<br><br>It produces blindness.<br>Foreign disease.

	Interview:<br><br>Do you know what disease you have in your eye? It isa disease that does not hurt anything, that does not burn, when a person has trachoma in a community, everyone can get sick from the same thing.<br><br>“The sight is damaged; a blind person is left alone; when you get old he can't see. That diseasecomes from another country.”<br><br>In the community of Villa Real, there is a prayer, the prayer cannot cure trachoma. Trachoma is not a disease, the disease is when you don't pray you fall ill, youget sick from animals- the whites tell us it's trachoma, but for us the disease falls<br><br>from not praying, eating food shot with a shotgun or with an arrow, produces eye disease. You have tosend to pray the food that was obtained by that means, the whites say it is trachoma. We do not suffer from trachoma in the Buenos Aires community, there is a Payé they pray for food, food.	It is not curable by the prayer.<br><br>Norm transgression culture (food).
**CATEGORY deductive**	**TESTIMONIALS**	**INDUCTIVE CATEGORY**
Concept of cleanliness and hygiene around<br><br>boys and girls	Alicia: “The child due to lack of care from the mother,the mucus sticks to the eyelashes and they become<br><br>infected, which is why the eye becomes sick. she emphasizes the responsibility of parents in caring for children.<br><br>Rosa: "The mother didn't bathe the child, that'swhy his head and face are rotting, that's why the child's eye is sick."<br><br>Rosa: "the child can have trachoma."<br><br>Luis": it is not good to have dirty children in general, because they eat with dirty hands it causes diarrhea and illnesses".	Gender roles (neglect ofthe mother).<br><br>Manifestation of the disease in childhood.<br><br>Lack of hygiene produces diseases.<br><br>Cleaning affects the goodgrowth of the child.<br><br>Bath, cleaning children.

	Alice: For the child to grow better, the child has to be clean, so that he doesn't grow weak.	Roles within the family and gender (sister care for younger brothers).
	Interview:<br><br>Personal hygiene-in children.<br><br>“You bathe them yourself, clean them, with cloth diapers, we buy them. when I came here the doctors gave me diapers”.<br><br>"The children of the community keep them clean, the indigenous people throw water on our faces, we crush the leaves of the forest and throw themon our faces, only the old wash their faces with leaves of the forest."<br><br>“When children are 7 years old, they already know how to wash their faces. The sisters are the ones who take care of the children, they are the ones with whom they wash their hands, their faces, one has to keep the children clean”.<br><br>"We bathe the grandchildren well, we take care of them."	Caregivers (grandmothers and grandparents).

CATEGORY deductive	TESTIMONIALS	INDUCTIVE CATEGORY
	"They throw water on their faces with both cupped hands, and without soap." "Washing with soap stick, you should not forget, one with those leaves has to wash hishead, to stay clean, wash clothes well"	
Education for health	Enrique: "I knew they arrived, but I was not in the community."<br><br>Luis: "They went to my community, they checked my eyes, they told me I had trachoma, the health promoter brought me, because they say I'm sick, and they also told other people that they also have trachoma, but that the others They bring tomorrow, because today they are celebrating and they can come until tomorrow; the whole community had their eyes checked."<br><br>Quiteria: "They did go to the community, but they didn't explain about trachoma."<br><br>Rogelio: "They explained about trachoma, with audiovisual help, about the disease, thetreatment. In the community we did translations so that the people in the community would understand".<br><br>"The commission has arrived twice, on both occasions the review has been carried out on all the people in the community."<br><br>Luis: "The health promoters provide education on hygiene, cleaning the home, some families do, especially women do not pay attention, some reject education. It is the woman's fault, because they are the ones who bathe the children and prepare the food".	Information to the community.<br><br>community (rituals, celebrations).<br><br>They visited the community, but did not explain the disease<br><br>Visit to the community withexplanation about the disease.
	Rosa: "People do pay attention to hygiene education, they bathe the children, they acceptlittle by little. they provide care when there is follow-up of the activities".<br>Enrique: "That they bring us medicines, because we need them; before it was good , because they gave education and cured people who got sick, in addition, Western medicine was used when the traditional one didn't work".	Gender roles regarding hygiene, cleanliness andfood preparation. Acceptance or rejection of the education provided by health promoters.<br>Lack of medicines. Before, the use of traditional medicine was respected.

Prevention	**How are children cared for in your community?**<br>"They have no knowledge of care, of hygiene, the culture is to raise them in the ground, leave them inthe ground while cultivating."<br><br>"Yes, trachoma occurs when the eyes are dirty,with rheum"<br><br>So that the children do not get sick, prayers aremade at meals, hunting and fishing.<br>María Helena: "Care guidelines for the different stages of development, as people grow they have to meet the traditional requirements to avoid getting sick." "Prayers are made to the foods (animals) that are hunted with a blowgun and arrow, they are theones that cause the most eye diseases, they whiten the eyes, these prayers are prevention."<br><br>**How to do to keep children clean?**<br><br>"When education on hygiene care is given, some families accept the recommendation,others do not, it is very difficult"<br><br>Quiteria: “My husband protects in what he knows, he prays to our children and grandchildren; they have not presented eye diseases. This protection is against serious diseases; If families prayerfully protect their children, diseases like trachoma would not occur, a disease that I hear about until now.”<br><br>“They have come to the community to check people with trachoma; The health workers went to pick up that woman, she felt pressured by her children and that is why she came to Mitú.”	Neglect of children in front of hygiene.<br><br>The disease is caused by alack of cleanliness in the eyes.<br><br>Protection throughprayer.<br><br>Standards Compliancecultures to prevent disease.<br><br>Protection in view of the disease through prayers.<br><br>Interference of health teams with daily routines – lack of timely information.<br><br>Grooming, house cleaning,contact with animals as a way to prevent disease.

	"Some people clean the children, others don't,they learn little by little."<br><br>**Does house cleaning help prevent trachoma?**<br>Enrique: "I don't know, it can give the disease, but I don't know how to respond."<br>"If you can give, playing with the dogs suddenly gets dirt in the children's eyes andcan cause the disease."	

	Rogelio: “I agree with cleaning the house, because uncleanliness can causeeye disease, one goes to the health post, but there are no medicines, even though there are nursing assistants.<br>Yes, eye diseases can occur, but so far we have no knowledge of that.<br>The ACAIPI organization has given information inthese times, that is why it is important to do the rituals for prevention, the time of the worm from June to July, the time when ophidian accidents occur; according to the criteria of ancestral medicine, protection is made.<br>Summer is from December to February, thereis also a time for crops and a time for the Yuruparí (fertility ritual), which is from March to April.<br>"Those from ACAIPI talk about the ecological calendar and have it immersed in the ethno- education plan."<br>Interview:<br>“To prevent, you have to cover the food, washyour hands, cover the pots; after going to thebathroom the flies eat excrement and come and bite you; flies bring disease, shit, worms, it's time to have clean hands before eating meals and lunches ".	Ecological calendar thatindicates the times and diseases that are present in them.<br>Food disposal and cleanliness to prevent illness.<br>The prayer cannot cure trachoma<br>The disease in the community does not existBecause cultural norms are met.
Announcement	Rogelio: "We already know how to do it, it is scheduled in advance, messages are sent with the people who go to the community where the message needs to be carried."	programming with anticipation.

	"When the meetings take a long time, people lose concentration, they are generally short, from 7 to 10 or 11 am, the meetings are alwaysheld and scheduled in the morning." "In general, the calls are made orally, or in the previous meetings that they have, the message is given."	Sending messages with someone who goes to the community.<br><br>short meetings for avoid deconcentration. Leveraging other meetings to deliver the message.
	"I listen to the radio station; Health messages arebroadcast by radiotelephone.<br>"In the Pirá area, all the communities communicate from 6 to 7 am with the community leaders."<br>In Santa Rosa del Pirá there is no radio; To inform the people of Santa Rosa, the ACAIPIorganization and the radio station communicate with Puerto Inayá, which is 5 minutes away, and they inform Santa Rosa".<br><br>"Through letters to the community or by radiogram, health information is transcribedand disseminated."<br><br>"Of the communities present, only Santa Rosa del Pirá, with 140 inhabitants, does not have radio communication."	Radio – station.<br><br>Office to the community or radiogram.
	Interview:<br><br>"The captain calls and notifies the meetings.The call is made voice to voice, a reason is sent.<br><br>"They live scattered, they live apart in a maloca, there is no radio."<br><br>When they get sick, where do they go?<br><br>"We are going to ask Santa Catalina for medicines, we live very far away."<br><br>"In Buenos Aires we have radio."	Voice to voice.<br><br>Dispersion as a barrier totimely communication.

Own resources in health	Sónaña, María Helena: "We attend the deliveryalone, we don't like to be helped". "Auxiliaries help to tend the delivery in some cases."<br>"Alto Pirá: Payés abound, there are 136 in totalaccording to the ACAIPI census".<br>"In Winambi there are no knowers."<br>"Santa Rosa / Pirá: prayers for thecures of the different times".	Payés, Cumú, specialistin prayer, dancers (knowledgeable about origin story and taught how to behave), healers, answering machines of dance (they participate in and maintain the rituals), healer of snake bite and sting of insect, healers with botany.
	"Puerto Inayá: there is an ancestral doctor totreat children and pregnant women."<br><br>"In Macaquiño there are no knowers (wise), theold are over."<br><br>"In Yapú there are knowers, dancers, healers, answerers of the dances-, they arepart of the work team of a traditional event(ritual), they are well organized in the traditional part."<br><br>"In Pirá, next to Santa Isabel and in Toaca,there are specialized people to deal with ophidian accidents (snake bites), it is a virtue of very few people."<br>"Insect bite: many people in the communityknow how to cure it."	
Health actions	1. "Identify children with eye diseases, eyes with rheumand runny nose."<br>Rogelio: "In the community, one does not pay attention to that, many times one leaves the responsibility to a public official." "Many times, one loses confidence in the official, because they do not pay attention to the notification made by the community."<br>"That the Ministry and the Secretary of Healthof Vaupés, educate the population and give medicines, because in the communities they have conjunctivitis."	Identification of children with runny nose and runny nose.<br><br>is delegated responsibility of Beware of public officials.<br><br>The notification is not made because the official does not pay attention to it – lack of recognition of the local actors.

	2."Announce to the Secretary of Health of Vaupés: how many people there are sick withvision by radio, to the Hospital, to ACAIPI".<br>3. "Indications to families how to keep children clean, teach siblings to wash their eyes, face and hands."	need for greater education and medicines by the officials and the sisters who care for the children.
water and sanitation essential	In Yapú: they drink rainwater. They refer to using soap to bathe andwash clothes, an exchange is made with schools and with other people toget the soap.<br>"She is in charge of bathing the children." "I havebeen living with my husband for 14 years, and he never bathes the children, I am the one who is watching over him."	Rainwater consumption.<br><br>Use of soap for bathing and washing clothes when there isenough to buy it with or when it is available.<br>Gender roles in child care.
	"We bathe with whatever we touch, Rey soap (a brand of laundry soap), bath soapor stick soap", that's how it is.<br>“The attendees report that it is so, you cannot have preferences; stick, vine or shell soap is used because it prevents gray hair. The ancestral soaps have the same function as the soap used by "the whites", it removesdirt, prevents gray hair." Antonio”: the use of plant soap has been lost, because now thereis commercial soap.”<br>Interview:<br><br>“We draw the water from the well, it is a clean puddle.”<br><br>“We buried the feces, they didn't leave us tanks, bathrooms, nothing. After July 20, thecaptain will request that."<br><br>"We draw water from the pipe."<br>"We throw the garbage anywhere, sometimeswe sweep, other times we sleep in a dirty house."<br>"There are almost no flies, in other houses I think thereare."	Use of traditional soaps<br><br><br>Well water consumption.<br><br>Disposal of excreta (burial).<br><br>Pipe water consumption.<br><br>Garbage disposal anywhere.

Mutual help	How do they help each other?<br>“We help to clean the chagra, we help to plant, to make chicha, we meet in the maloca, those of the community, we all helpeach other to clean.<br><br>Enrique: "It's not good for adults to beblind."<br><br>Moisés: "When the family is going to do work in their chagra, they invite the other families and prepare in advance, going fishing, hunting, preparing food for the guests who are going to help them." “*came yedoye*” (Piratapuya language).	It helps to clean the chagra, sow, make chicha, cleaning the maloca.
	Saint Catherine. “If we have cassava, there areno fish; sometimes animals are hunted, one goes far away and hunts (it takes about a day).They live in the caño agua blanca.	dispersal, resources

Deductive: from where conceptual frameworks that go from the general to the particular and the **inductive.** They arise from the information that is collected and go from the particular to the general.

Matrix 2.

CATEGORIES deductive	SUBCATEGORIES	INDUCTIVE CATEGORIES
Knowledge about the trachoma disease	Explanation	Curse caused by envy.<br><br>Transgression of the cultural norm,related to food (from the symbolic).<br><br>inexplicable mystery.<br><br>Presence of diseases according to seasons(ecological calendar).<br><br>Alien disease.
	Recognition	It can cause blindness, produces blindness(trachomatous trichiasis).<br><br>Inverted eyelid (trachomatous trichiasis).<br><br>It can be submitted at any time.<br><br>There is no pain or burning.<br><br>contagious
	Prevention	Neglect of children's hygiene.<br><br>The disease is caused by a lack of cleanliness in the eyes.<br><br>Protection through prayer.<br><br>Compliance with cultural norms to prevent disease.<br><br>Cleaning, cleaning the home, contactwith animals as a way to prevent thedisease.<br><br>Ecological calendar that indicates the timesand diseases that occur in them.<br><br>Food disposal and cleanliness to prevent illness.<br><br>The rezandero cannot cure trachoma.

CATEGORIES deductive	SUBCATEGORIES	INDUCTIVE CATEGORIES
		Illness in the community does not exist because cultural norms are met.<br><br>Before, the use of traditional medicine wasrespected.
	Treatment	Incurable disease in some cases.<br><br>White dots in the eye, treated with traditional plants, while trachoma has nocure.<br><br>It is not curable by the prayer.<br><br>As a way to avoid pain, the eyelashes areremoved when the eyelid is inverted.<br><br>Lack of medicines.<br><br>Before, the use of traditional medicine wasrespected.
Concept of cleanlinessand hygiene around boys and girls		Gender roles (neglect of the mother). Manifestation of the disease in childhood.Lack of hygiene produces diseases.<br>Cleaning affects the good growth of the child.<br><br>Bath, cleaning children.<br><br>Gender roles regarding hygiene, cleaningand food preparation.<br>Gender roles (sister care for younger brothers).<br><br>Caregivers (grandmothers and grandfathers).

Education for health	forms of communication	Information to the community.<br><br>Interference of health teams with daily routines – lack of timely information.<br><br>Times - community cycles (parties, celebrations).
**CATEGORIES deductive**	**SUBCATEGORIES**	**INDUCTIVE CATEGORIES**
		Visit to the community, but no explanation ofthe disease.<br><br>Visit to the community with explanationabout the disease.<br><br>Acceptance or rejection of educationprovided by health promoters.<br><br>Programming in advance.<br><br>Sending messages with someone who goesto the community.<br><br>Short meetings to avoid deconcentration.<br><br>Leveraging other meetings to deliverthe message.<br><br>Radio – station.<br><br>Office to the community or radiogram.<br><br>Voice to voice.<br><br>Dispersion.
Own resources in health		Labor assistants (mother-in-law, husband, sister, grandmother, mother), peasants, prayers, dancers, healers, dance answerers,snake bite and insect bite healers.

Health actions		Identification of children with runny nose andrunny nose.<br><br>Responsibility for care is delegatedto public officials.<br><br>The notification is not made becausethe official ignores it – lack of recognition of local actors.<br><br>Need for more education and medication on the part of officials.
community fabric		Cleaning the chagra, sowing, making chicha,cleaning the maloca.
Context	Water and basic sanitation	Rainwater consumption.

**CATEGORIES deductive**	**SUBCATEGORIES**	**INDUCTIVE CATEGORIES**
		Use of soap for bathing and washing clothes.<br><br>Use of traditional soaps.<br><br>Well water consumption.<br><br>Disposal of excreta<br><br>(burial).Pipe water<br><br>consumption.<br><br>Garbage disposal anywhere.
	Geographic location	Dispersion
	Food safety	Consumption of cassava, game animalsand decrease in fish.

PART V

ANALYSIS OF THE RELATIONSHIP OF TRACHOMA WITH THE CULTURE OF THE INDIGENOUSPOPULATIONS AFFECTED IN THE DEPARTMENT OF VAUPÉS

1. Knowledge about Trachoma

1.1. Explanation: A tendency was expressed on the part of the majority of the focus group participants to interpret the disease as a transgression of the cultural norm, related to the consumption of prohibited foods atcertain times framed in the ecological calendar and with curses. Here are some testimonials that support this statement:

> *"...this disease is not normal; the trachoma disease is a curse that the same people create. In some communities there is a lot of envy for what they have, that's why we hurt ourselves..."*

> *"… sickness is when you don't pray, you get sick, you get sick from animals - the whites tell us it's trachoma, but for us we get sick from not praying, eating food caught with a shotgun or arrow, itcauses eye disease. The food obtained by this means must be sent to pray, the whites say it is trachoma. We do not suffer from trachoma, in the Buenos Aires community there is a Payé, they pray for food…"*

> *"...in addition, there are several cultures that do not take care of the culture, when they eat mojojoy from the pupuña palm or those that have thorns, they produce trachoma disease. The palm mojojoy that do not have thorns can be eaten..."*

> "... conjunctivitis and tooth pain according to the Payé occurs during the peach season and the time of the tapurú worms..."

However, it is important to consider that in the focus group, 18% (represented by 3 people), with a higher levelof education and one with experience as a health promoter, considered that trachoma is a foreign disease or a Western disease.

Likewise, it must be recognized that there is an ecological calendar, which determines the presence of diseases according to both traditional and western times of the year.

The calendars are an expression of the organized practices of the peoples, according to different times of the year, and their constellations, on which the increase or decrease of food, the presence of diseases, the indicated moments for holding festivals, depend. rituals and celebrations, rainy and dry seasons, among other aspects. Hence, the calendars can be differentiated into agroecological and food calendars, festivities, rituals and epidemiological.
Regarding the ecological calendar and the relationship with diseases, the following was mentioned in the focus group:

> *"...the ACAIPI organization has obtained information from these times, that is why it is important to carry out the rituals for prevention, the time of the worm from June to July, a time when ophidian accidents occur, according to the criteria of traditional medicine, the protection. Summer, December to February, growing season. Yuruparí season, March to April. Those from ACAIPI talk about the ecological calendar and have it immersed in the ethno- education plan..."*

> *"...In the time of the worm it is the most dangerous time, because it produces many diseases and they protect themselves in a spiritual way, through tobacco and breo...*

Ecological calendar

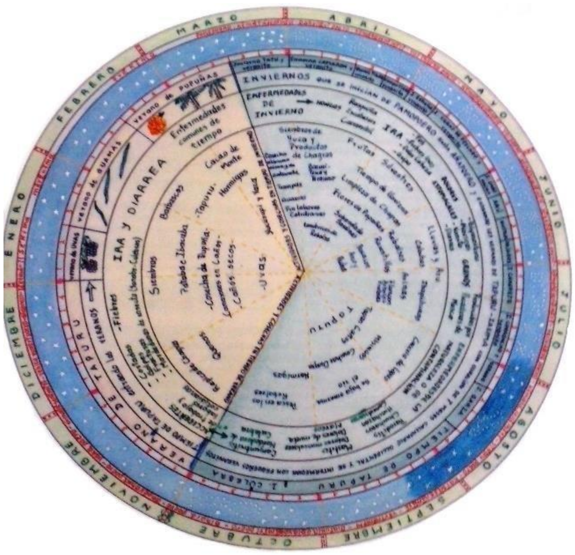

Photo No. 10 Taken from the text Intercultural Basic Care Plan of the Anthropologist Juan Guevara Garzón, Administrative Department of health of Vaupés, 2003.

1.2. Recognition: The recognition given to trachoma as a Western disease has been influenced by the dissemination of information through visits made by the health institution. However, it is evident that, in the majority of focus group participants, Western knowledge has not been very internalized. And the explanations derived from his worldview continue to prevail, this is expressed in the following testimony:

> *"...in addition there are several cultures that do not take care of the culture, when they eat mojojoy from the pupuña palm or those that have thorns, they produce trachoma disease. The palm mojojoy that do not have thorns can be eaten..."*

They recognize that the disease can cause blindness, that it is a contagious disease, although they are not clear about how it is contagious, and even less that it is associated with cleanliness, particularly of the face. The participants also do not relate the symptoms (when they say that it is a disease that they do not have, pain or burning), they are not clear about the difference between acute and advanced signs. Trachoma is recognized to occur at any time of the year. when it was done the mirror exercise expressed that this was a useful object, to be able to remove the eyelashes that are inside the eye, however, they do not link the discomfort of the eyelashes with trachoma and blindness.

1.3. Prevention: Focus group participants handle a highly internalized concept of prevention and protection related to their culture. For them, prevention is directly related to protection through cultural practices consisting of the prayer proffered by P*ayé* either *Cumu.* This is how they express it in their testimonies:

> *"...The community has a concept of disease prevention marked in its worldview, expressed in the continuous consultation with the Payé and the Cumú, prior to carrying out:*
>
> *1. Any daily activity: fishing, hunting, building chagra, going to sacred mountains or going to another community.*
>
> *2. Changes in growth and development stages:*
>
> *Gestation: protection of the baby and arrangement of the womb, as well as the prayer to the diet and recommendations regarding the food that should be consumed.*
>
> *Birth: El Payé, Cumú performs protection of the child by painting it with carayurú or prayed tobacco.*
>
> *Weaning: The prayer of the food complementary to breastfeeding is performed.*

First menstruation: Since it is a time of risk for women, pray to protect her and a special diet isrecommended.

“In addition to the protections listed above, there are more comprehensive protections related to the environment and people in the communities, including eye protection; To avoid eye diseases, the Payé recommends "...Do not eat worms that breed in palms that have thorns such as the pupuna, the corombolo...".

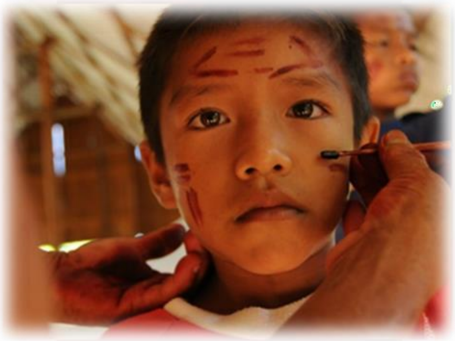

Photography No 11 carayurú-red face-protection against diseases
Source: Prezi presentation, by Francy Luz Uribe and Mauricio Cárdenas

It should be noted that there is no concept of disease prevention both own and western from the perspective of allopathic medicine. Prevention from the Western perspective is achieved when there iscontinuous and systematic monitoring by health personnel:

“People do pay attention to hygiene education, bathe children, they accept little by little. They provide carewhen there is monitoring of the activities.”

The ecological calendar determines the time in which diseases must be protected and prevented. Examples: the time from mid-March to the end of October, are defined as winter and correspond to constellations such as: whole shrimp,whole tiger, star, yerado. of the fish, whose diseases of this season are fungi, which produce itching, scabies, carranchil, respiratory infections and stomach pains. Conjunctivitis appears at the end of October in the constellation of the snake. (see diagram of the Ecological calendar of the Tucano Oriental indigenous people: (Takenfrom the text Basic Care Plan1Intercultural, Juan Guevara Garzón year 2003).

The constellations that were previously stated and that are included in the ecological calendar refer to the shapes thatthe stars take at different times of the year, which they relate to their mythical animals, such as the tiger, shrimp, snakes, otter, the fish, armadillo or tattoo, etc., as can be seen in the following photographs:

Constellation of the Snake.

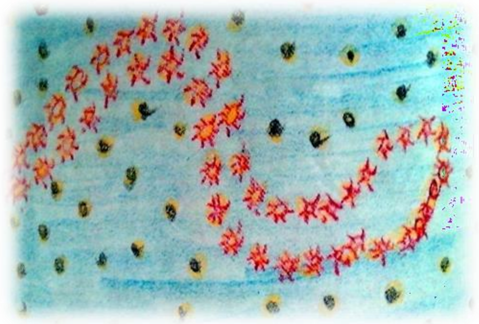

Constellation son of Ñapiarato.

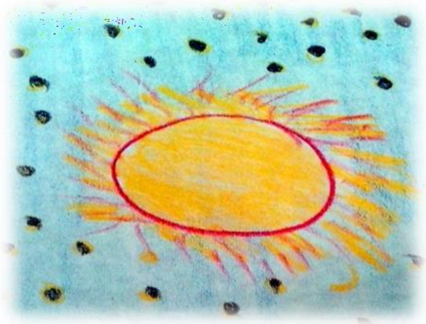

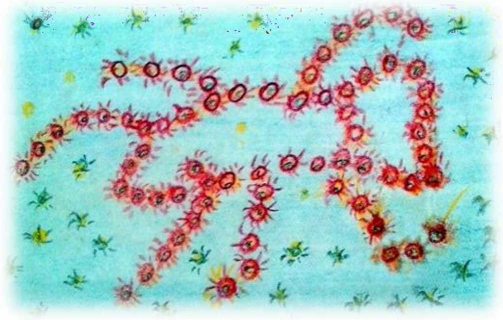

Shrimp constellation

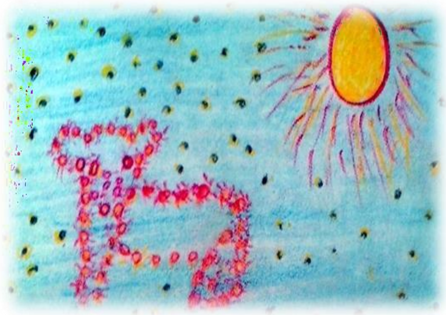

Constellation of the Tiger.

1.4. Treatment.

The testimonies of the participants in relation to the cure are diverse, for some like Yujup they consider that itis a malefic disease that cannot be cured by traditional medicine, although sometimes by Western medicine. Testimonial below:

> *"...This disease is not normal, the trachoma disease is a curse that the same people create." in somecommunities there is a lot of envy for what they have, that's why we hurt ourselves". "This disease is incurable for us, when the disease starts in children it causes blindness, sometimes doctors cure the disease, but in other cases they don't...".*

On the other hand, others like the Tucanos consider that blindness due to evil is different from blindness caused by trachoma and thatit cannot be cured with traditional medicine.

Testimony

> *"...all diseases are called eye pain, but when white dots occur, when vision is lost, it is calledwhite dot eye. But that has its cure, we have tried with traditional plants", but this disease- trachoma- is different, it has no cure...".*

1

The Yujup of Santa Catalina do not recognize pain or burning as symptoms of trachoma, this non- recognition of symptoms may be related to repetitive infections, becoming normal in daily life, therefore, they do not undergo any treatment, nor consult western medicine. In the trichiasis phase, the eyelashes are removed with tree wax, as a measure that reduces discomfort inside the eye. Other reasons why they do not consult are because there are no permanent health personnelin the rural area of the department of Vaupés, and where there are, they do not have medicines, and there are also geographical access barriers to the municipal capitals, which makes timely consultation difficult.

Testimony:

"You go to the health post, but there are no medicines, even though there are nursing assistants."

1. Concept of cleanliness and hygiene around boys and girls

2.1. Gender role and family. The concept of cleanliness is closely related to gender and family roles, for example, menmake women responsible for caring for children, even these since childhood, are in charge of caring for their younger siblings. Regarding the family role, young women for work reasons usually leave their children in the care of grandparents, uncles, aunts or other relatives. It is common today to arrive in a community and find families made up only of grandparents and grandchildren.

2.2. Hygiene and Cleaning Concept. The participants recognize personal hygiene and cleanliness of the house as a disease prevention measure, however, these preventive measures are not part of their daily practices, which may be associated with the productive activities they carry out such as fishing, hunting, gathering, planting cassava, which forces them to spend most of the day outside the house. The concept of hygiene is more related to a concept of spiritual cleanliness and they have the habit of taking a morning bath to revitalize their energies, but not so focused on personal hygiene.

Of all the participating ethnic groups, the Yujup are the ones that apply the concept of cleanliness and basic sanitation the least. Possibly because they are a semi-nomadic people, they have a transitory vision of housing and territory. A large part of their daily life is spent in the mountains, collecting the harvests of the environmental offer.

The non-existence of intra-domiciliary drinking water negatively affects the practice of personal and family hygiene, evidenced in the collection of water in places far from the house, which implies a rational use of thatwater, basically for food preparation.

The communities in rural areas that are more dispersed or far from populated centers do not have the means to supply themselves with cleaning products, either for economic reasons or for their availability. In the absence of such products, theyuse those offered by the environment, such as bark or leaf soap, which fulfills the same function as the soaps available in the market.

> *"...we indigenous people throw water on our faces, we crush the leaves of the forest and throw them on our faces, only the old wash their faces with leaves of the forest...".*

2.3. Relationship of Hygiene and Cleanliness with childhood illness

According to testimonies of some participants, the lack of cleanliness in children is one of the manifestations for the appearance of diseases, reflected in the slow growth and development of the minor, their parents and caregivers areresponsible for this condition. It should be considered that many of the ethnic groups show resistance to the recommendations on cleanliness and hygiene by health promoters, some testimonials that support this statement:

. . . They have no knowledge of care, of hygiene, the culture is to raise them in the ground, leave them in the ground while cultivating...”

“. . . It is not good to have dirty children in general, because they eat with dirty hands, it causes diarrhea and illnesses...".

"...for the child to grow better, the child has to be clean, so that he does not grow weak..."

“...in the child, due to lack of care from the mother, the mucus sticks to the eyelashes and they become infected, which is why the eye becomes sick. Emphasizes the responsibility of parents in caring for children...".

"...care guidelines for the different stages of development, people as they grow have to meet the traditional requirements to avoid getting sick...".

"...When hygiene care education is given, some families abide by others not, it is very difficult..."

3. Health education

3.1. Information and education to the community

Communities are receptive to health days or activities as long as they are notified in advance and can be organized, so as not to interfere with their individual, family, and community scheduled daily activities. Among the participants, there are those who acknowledge the visit by health personnel and the information that has been given about trachoma, as there are those who say they have not been visited or argue that the information provided is not clear or sufficient, or that they have gone, but not They have given you the information. The promoters who participated in the focus group argue that giving education in relation to personal hygiene or cleanliness is difficult due to the lack of receptivity or inconvenience on the part of the families,

"...I knew they arrived, but I was not in the community..."

“...they went to my community, they checked my eyes, they told me I had trachoma, the health promoter brought me, because they say I'm sick,

“the whole community had their eyes checked”. “if they went to the

community, but did not explain about trachoma...".

"...they explained about trachoma, with audiovisual help, about the disease, the treatment. In the community we did translations so that the people in the community would understand..."

"...*The commission has arrived twice, on both occasions the review has been carried out on all the people in the community".*

"Health promoters provide education on hygiene, cleaning the home, some families do, especially women do not pay attention, some reject education..."

"...it's the woman's fault, because they are the ones who bathe and prepare the food..."

3.2. Communication

One of the forms of communication between indigenous groups is given mainly by "voice to voice" and radiogram in the towns where they have radio. Another of the means that some communities near the urban area of Mitú have, is the radio, since, through the stations present, such as the Community and the Army, the announcements are issued and the calls are made for the visits of the personnel of health. On some occasions, boats or plane trips are used to send written notes to communities that do not have the aforementioned means. There is also the use of other own meetings to deliver the messages.

The community meetings that the health personnel are going to hold must be consulted and arranged, the participants emphasize that they must be held in the morning hours and in short shifts so as not to lose concentration, they must also be more constant so that the community keeps alive knowledge and it can be reinforced, others reported that they have been given information but that they have forgotten. It is important to consider the times and community cycles (parties, celebrations, so as not to interrupt them and in turn ensure that the visit is effective). The festivals and celebrations are generally carried out at harvest and fish harvest times, for example: the harvest of pupuña[2] in the months of December and January summer time of Grapes, harvest of wansoco[3] and wasay[4] which is from the middle of March to the beginning of April and the winter time tatú[5]. In the month of April is the rise of fish that is related to the constellation of winter shrimp, tiger beard and tiger body.

---

1. Pupuña: Chontaduro is the same as in other regions.
2. Wansoco: it is a wild fruit typical of the Amazon region with a viscous and sticky content.
3. Wasay: it is a fruit equally typical of the region that is known in Brazil as açaí.
4. Tatú: the armadillo.

3.3. Interference of health teams with daily routines – lack of timely information.

One of the great difficulties reported by patients and ex-patients is the lack of articulation between the different institutions, such as the health provider entities (Caprecom - Mallamás), which did not notify them in time on the last surgical day, resulting in the absence of many people suitable for surgery, others had to leave their chores halffinished. They argue that they should be notified in advance to be able to keep their personal, family and community affairs up to date, as well as to carry out the pertinent protection.

> *"...they also told more people that they also have trachoma, but that they bring the others tomorrow, because today they are celebrating and that they can come until tomorrow...".*

4. Health actions

4.1. Own health actions. Own health actions are related to ecology, hence the ecological calendar is born, which has several components: the constellation, related to the seasons of weather and harvest. Health actionsare fundamentally preventive to avoid ecological and social imbalances, for that there are cultural norms such as the definition of sacred sites, diets according to the life cycle, to the diseases that occur, to the times of the ecological calendar, prayers of protection for the development of daily activities, for a good harvest and for theprevention of diseases, and burning of breo for the protection of the territory from spirits of nature.

When the disease occurs, it is also treated mainly with prayers, blows to the sick person and/or family or to what is going to be taken, and intake of medicinal herbs, according to the recommendations given by the Payé, Cumú or Blower. For delivery care, the women in the focus group state that they attend delivery alone, they do not like to behelped.

> "Auxiliaries help to tend the delivery in some cases."

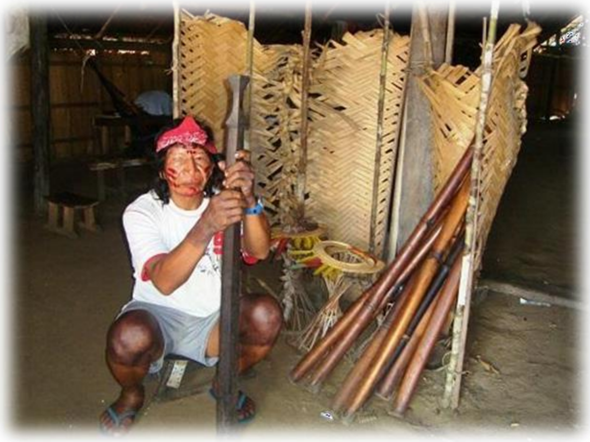

Photo No. 13 Payé from the Pirá Paraná area

4.2. Own resources in health

Some of the own resources that indigenous communities have are: human resources such as the Payé who is the ancestral knower, guides the community in the periods of hunting and planting, is the guardian of food, solves problems interpersonal, restores the social or natural balance when it is broken, maintains traditions and participates in actions to prevent and treat diseases. The Cumú is another own resource in health, who manages several aspects: sucks the disease locating the affected site and prays based on the diagnosis found, performs ceremonies and rituals of traditional education, sometimes replaces the Payé. They also have the blower, who is a lower-category healer, learns his procedures from an elder in the group, without requiring any formality, the blower is not aggressive and there is no rivalry between them. There are other specialists incharge of dealing with ophidian accidents and insect bites.

The other own health resource is botany, which consists of the use of medicinal plants used by the Payé,some for diagnosis, others for cures and others for prevention.

Testimonies reported by the participants in relation to the situation of their own health agents according to theareas of origin:

> *"---Alto Pirá: the Payés abound "there are 136 in total. By ACAIPI census. Winambi: There are no knowers.*
> *Santa Rosa/ Pirá-Paraná: there are Prayers for cures at different times. Pto. Inayá: there is a traditional doctor to treat children and pregnant women. Macaquiño: there isn't, the old ones are over.* Yapú: there are experts, dancers, curators, answerers of the dances-they are part of the work team of a traditional event, they are well organized in the traditional part "-For the sides of Santa Isabel and Toaca, there are specializedpeople to attend to the accident ophidian, is a virtue of very few people. Insect bite: many people in the community know how to cure…"

5. community social fabric

The basis of society for the indigenous peoples of Vaupés is based on what each individual contributes to thecommunity, both in the family fabric and within their community. Basically, the social fabric is framed in community activities such as the construction of the chagra and maloca, preparation of the chicha for celebrations or community meetings, cleaning of the maloca and common places, for the preparation of rituals, where the dancers6and answering the dances7, in traditional events such as Dabucurí and Yuriparí[8].

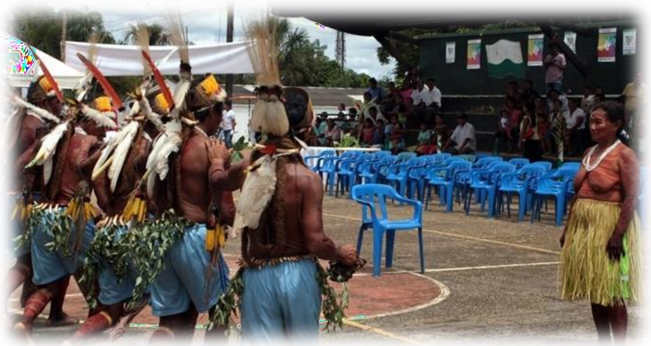

Photo No.14.**dance answering machine**

[6]The Dancer: His dance is related to the growth and fertility of everything found in nature.
[7]Dance answerers: She is part of a group of knowers, she is the owner of the maloca, the mother of the crops and the owner of the chagra. She is in charge of organizing and encouraging the women to prepare the chicha for the party. She accompanies the dancers

singing responses to certain passages of the verses of the dances.

[8]Yuruparí: it is a ritual festival of passing from boy to man, only men participate. It initially consists of making a diet based on Manivara (ant) and water.

In some communities they hold meetings in the morning where they share food prepared by each of the families attending, as is the case in the communities of the Querarí and Pirá-Paraná areas, among others.

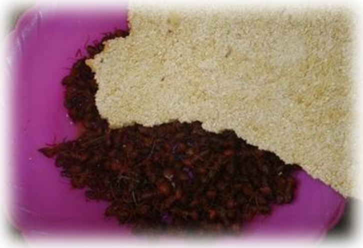

Photo N0 15: Manivara ant, used in the Yuruparí ritual.

6. Proposal for community surveillance for trachoma.

At the end of the day, the focus group discussed the responsibility of the different parties regarding the prevention of the disease and concluded that this is the responsibility of the health entities, since they are the ones that must provide, according to them, the guarantees for avoid trachoma. However, they expressed their support for the actions that institutions must carry out, with the identification of children with runny nose and runny nose, as well as people who do not have eyelashes, who are blind or have some other eye problem. In addition to the receptivity to the medicines that are distributed within the framework of the SAFE strategy.

Another of the conclusions reached by the focus group was that a channel should be established within the community for the notification of possible cases to the different health institutions. He was also willing to work with families in cleaning both personal, face, and homes, and they hope that they will have a continuous process, accompanied by educational exercises, supply of medicines, and health personnel. As part of the educational process they proposed to make messages alluding to prevention in their language as shown in the following photograph.

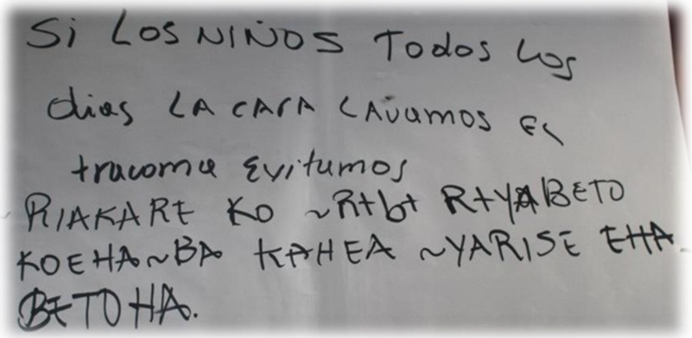

Photo No. 16 Message to prevent trachoma in the Toucan language

The development of the focus group was carried out within the framework of the third surgical day, for patients diagnosed with trachomatous trichiasis. The participants prepared a message of thanks to the entire team that supported this process. The photograph below shows the message both in language and its translation in Spanish.

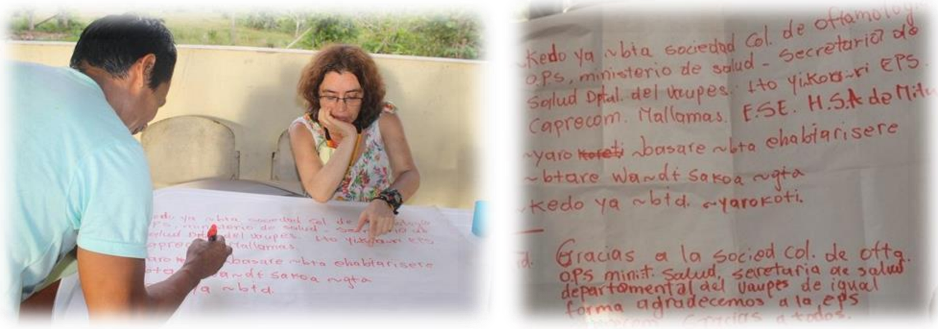

Photos No. 17 Thank you messages in language and Spanish

PART VI

CONCLUSIONS

1. As referred by the participants, trachoma is a disease that has been known for several years, since there is traditional knowledge related to practices and consumption of traditional foods, which may show that it isan event that occurs in these communities. from time ago.

2. Participants clearly identify acute eye diseases such as conjunctivitis. The recognition of diseasesrelated to the symptoms of the advanced stages of trachoma is not clear.

3. Conjunctivitis is clearly located at a specific time in the ecological calendar. Apparently, the symptoms of theadvanced stages are related to the inadequate consumption of food at times that are not allowed.

4. Some of the participants with a higher level of schooling identify conjunctivitis as a contagiousdisease.

5. Despite the fact that the participants more or less clearly identify the symptoms of the acute forms and the advanced formsof trachoma, they do not relate it as the same disease and that it is the natural history of the same.

6. For the participants, the concept of prevention and treatment of diseases is linked to the traditional concept, to thepractices and recommendations defined by the traditional authority, to the follow-up of traditional norms and to compliance with the ecological calendar.

7. A clear idea of western treatment for trachoma is not identified within the participants.

8. The concept of hygiene is related to gender, giving relevance to practices carried out by women. It is identified as atraditional spiritual practice linked more to the revitalization of energy than as an external cleansing of the body and face. However, it is assumed as a daily practice, but it is recognized that it is not performed frequently.

9. Participants do not associate the state of hygiene and cleanliness of the face with trachoma or as a form oftransmission.

10. Voice-to-voice, radio and radio were identified as effective means of communication in indigenous communities(the latter with restrictions due to unavailability in some communities).

11. Despite the fact that the activity was carried out with different peoples with different languages and that there were communication difficulties, it was possible to maintain attention and participation with great receptivity until the end ofthe day.

RECOMMENDATIONS

1. Carrying out a focus group in several languages was a limitation for the understanding of the testimonies, at times it was not clear to us if the translator had conveyed the precise message as expressed by the participant or what the moderator wanted to know, so which suggests that new activities of this type be carried out by indigenous peoples.

2. In the development of the focus group, very valuable testimonies were collected that must be considered in the construction of the Information, Education and Communication strategy, from their worldview (ecological calendars, rites of passage, myths of origin, rituals), forms of communication, social fabric, forms of health care and own resources in health.

3. The ecological calendar is highly internalized in the participants, conjunctivitis is located in a defined time of the calendar, so it is important to take this time into account for the intensification of education and communication actions. It is also important to arrange the inclusion in the ecological calendar of prevention actions and that this be used as part of the IEC strategy.

4. In the design of IEC strategies, traditional knowledge must be incorporated, as well as existing traditional and community resources.

5. It is considered necessary for the IEC strategy to be systematic, constant and elevated to a public policy so that it has continuous financing and does not depend on temporary agreements, positively impacting the national achievement of the elimination of trachoma.

6. Definitely there must be prior consultation and agreement with the communities, for the effective development and achievement of the programmed health actions, and not interfere with the activities of the community.

7. Trachoma should rise as a topic of interest in the agendas of both the health sector and the different local, regional, departmental, national and international actors, for the operation of community surveillance and the development of components F and E of the SAF strategy.

8. It is necessary the presence of health personnel in the field trained in pedagogical strategy, to achieve the acceptance of the community and to develop the IEC strategy permanently, generating behavioral changes and re-signification of the hygiene concept of the communities affected by the trachoma

9. The Yujup indigenous people is the one that presents the greatest problems in all issues, making it the most vulnerable and endangered indigenous community, for which it is necessary to deploy an urgent intervention.

10. It is important to have the support of the education sector to achieve the deployment of the IEC strategy, in the school environment.

11. For the formulation of a community surveillance strategy, the channels and flow of information must be clearly identified in advance and this must support the institutional surveillance strategies.

12. In one of the texts by Juan Guevara Garzón, he proposes working as the central axis of Primary care community, "Water" and proposes to do it from two perspectives, one would be: water quality, water use and its relationship with health and the other perspective Water and History, (the before, the current and future state of this).

DE INVESTIGACIÓN DESARROLLO E INNOVACIÓN

COMITÉ DE ÉTICA EN INVESTIGACIÓN

Bogotá, Julio 10 de 2014

Doctor
JULIAN TRUJILLO TRUJILLO
Coordinador Programa Nacional de EID
Ministerio de Salud y Protección Social
La ciudad

Apreciado Dr. Trujillo

El Comité de Ética en Investigación (CEI) de la Escuela Superior de Oftalmología del Instituto Barraquer de América, en reunión ordinaria, consideró que el Proyecto **"CONOCIMIENTOS, ACTITUDES Y PRÁCTICAS DE HIGIENE Y PREVENCIÓN DEL TRACOMA OCULAR EN LA POBLACIÓN INDÍGENA DEL VAUPÉS, AMAZONÍA** COLOMBIANA", reúne los requisitos éticos mínimos para su aprobación, por lo cual se da el aval ético según acta 07 del 10 de Julio de 2014.

En caso de ser modificada la versión del proyecto avalado por el CEI, con relación al componente ético, se solicita su colaboración para informar al Comité para su respectiva revisión.

Cordialmente,

Clara B. López de Mesa M.

CLARA BEATRIZ LOPEZ DE MESA MELO
PRESIDENTE COMITÉ DE ETICA EN INVESTIGACIÓN
Escuela Superior de Oftalmología
del Instituto Barraquer de América
